# Antibody–Drug Conjugates: The Last Decade

**DOI:** 10.3390/ph13090245

**Published:** 2020-09-14

**Authors:** Nicolas Joubert, Alain Beck, Charles Dumontet, Caroline Denevault-Sabourin

**Affiliations:** 1 GICC EA7501, Equipe IMT, Université de Tours, UFR des Sciences Pharmaceutiques, 31 Avenue Monge, 37200 Tours, France; caroline.denevault@univ-tours.fr; 2 Institut de Recherche Pierre Fabre, Centre d’Immunologie Pierre Fabre, 5 Avenue Napoléon III, 74160 Saint Julien en Genevois, France; alain.beck@pierre-fabre.com; 3 Cancer Research Center of Lyon (CRCL), INSERM, 1052/CNRS 5286/UCBL, 69000 Lyon, France; charles.dumontet@chu-lyon.fr; 4 Hospices Civils de Lyon, 69000 Lyon, France

**Keywords:** antibody–drug conjugate, ADC, bioconjugation, linker, payload, cancer, resistance, combination therapies

## Abstract

An armed antibody (antibody–drug conjugate or ADC) is a vectorized chemotherapy, which results from the grafting of a cytotoxic agent onto a monoclonal antibody via a judiciously constructed spacer arm. ADCs have made considerable progress in 10 years. While in 2009 only gemtuzumab ozogamicin (Mylotarg^®^) was used clinically, in 2020, 9 Food and Drug Administration (FDA)-approved ADCs are available, and more than 80 others are in active clinical studies. This review will focus on FDA-approved and late-stage ADCs, their limitations including their toxicity and associated resistance mechanisms, as well as new emerging strategies to address these issues and attempt to widen their therapeutic window. Finally, we will discuss their combination with conventional chemotherapy or checkpoint inhibitors, and their design for applications beyond oncology, to make ADCs the magic bullet that Paul Ehrlich dreamed of.

## 1. Introduction/History

Antibody–drug conjugates (ADCs) have made considerable progress in 10 years. ADC is a vector-based chemotherapy that allows the selective delivery of a potent cytotoxic agent within a tumor. An ADC results from the generally stochastic grafting of a cytotoxic agent onto a monoclonal antibody (mAb) via a judiciously constructed spacer arm [1,2]. This is a complex mixture of immunoconjugates with different DLD (drug loading and distribution) and DAR (drug-to-antibody ratio, corresponding to the number of cytotoxics grafted onto the mAb) [3]. In 2009 gemtuzumab ozogamicin (Mylotarg^®^) was the only ADC approved by the Food and Drug Administration (FDA) and 12 other candidates were in clinical studies [4]. At present, 8 other ADCs have been approved and more than 80 others are in active clinical studies, including 6 in phase III or pivotal phase II (Table 1) [5]. More than 50 candidates have also been abandoned in the clinic mainly for toxicological reasons or because of a lack of efficiency. ADCs targeting solid tumors are currently making a satisfactory breakthrough in the clinic. Until November 2019, only Kadcyla^®^ had an indication in solid tumors. With the late 2019 FDA-approval of both Padcev^®^ and Enhertu^®^, and Trodelvy^®^ in April 2020, there are currently four FDA-approved ADCs directed against solid tumors. The other five ADCs are indicated in hematological cancers and are generally considered to be easier to target with ADCs. In addition, ADCs in the advanced clinical phase are mainly directed against solid tumors (four against solid tumors; two directed against lymphomas).

In 2009, calicheamycins, auristatins and maytansinoids were the main classes of cytotoxics used for ADC development. Ten years later, these same molecules are still used among other payloads optimized for better stability and hydrophilicity. New classes of cytotoxics have also been developed (PBDs, duocarmycins and camptothecin derivatives). In 10 years, considerable progress has been made in antibody engineering to allow more site-specific conjugation, to improve the homogeneity and the stability of the constructions and to bring 2nd and 3rd generation ADCs to the clinic in the hope to broaden the therapeutic index (ratio of the median lethal dose (LD_50_) to the median effective dose (ED_50_)) [1]. Several dozen bioconjugation technologies based on cysteine residues, non-natural amino acids or patterns introduced by molecular engineering have been proposed in preclinical studies [6]. Finally, more tumor specific antigenic targets and optimized release mechanisms of the cytotoxic agent within the tumor have led to the development of more performant ADCs [7,8,9].

This review will focus on FDA-approved and late stage ADCs as well as their limitation including their toxicity and associated resistance mechanisms. We will describe new emerging strategies to deal with these issues, including 3rd generation molecular constructions, the choice of alternative vectors, innovative delivery systems and combinations of ADCs with conventional chemotherapy or immune checkpoint inhibitors.

## 2. Design, Mechanism of Action and Therapeutic Indications of FDA-Approved First- and Second-Generation ADCs

**Figure 1:**
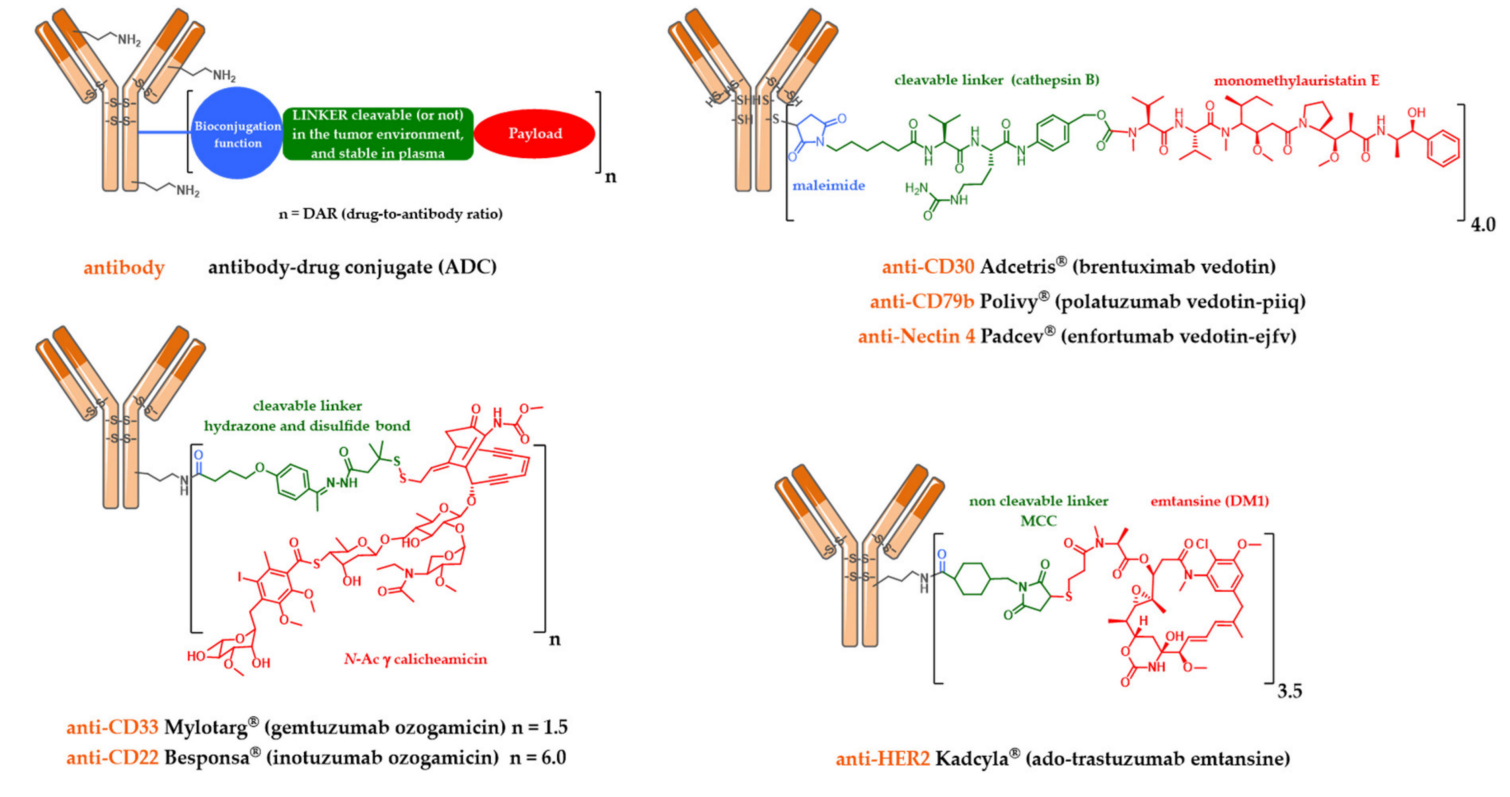
Schematic representation of the first and second generation FDA-approved ADCs: Mylotarg^®^, Adcetris^®^, Kadcyla^®^, Besponsa^®^, Polivy^®^ and Padcev^®^.

The development of immunoconjugates in oncology has enabled the emergence of two key elements necessary to ensure the success of an ADC. The first concerns the need of a linker between the mAb and the payload. This mAb-linker-payload system was first designed with a cleavable linker (Figure 1) assumed to be stable under physiological conditions during plasma circulation, and quickly cleaved after tumor cell endocytosis, in order to selectively deliver the payload in the tumor and limit the appearance of undesirable side effects due to off-target toxicities. This type of linker is sensitive to lysosomal conditions (proteases, acidity and a reducing medium). The second key element for an ADC is correlated with the necessity to have a powerful cytotoxic agent grafted to the antibody. Indeed, the first ADCs were characterized by a low therapeutic index due to the low potency of the payload (e.g., anthracycline), resulting in a very limited therapeutic effect at the maximum tolerated dose (MTD) of 100 mg/kg. This phenomenon can be explained by the low antigenic density on the cancer cell surface and by the small percentage of ADC able to reach the tumor cell surface compared to the injected quantity (hardly exceeding 0.1%) [10].

### 2.1. Mylotarg^®^, Besponsa^®^ and the First-Generation Cleavable Linker

**Scheme 1:**
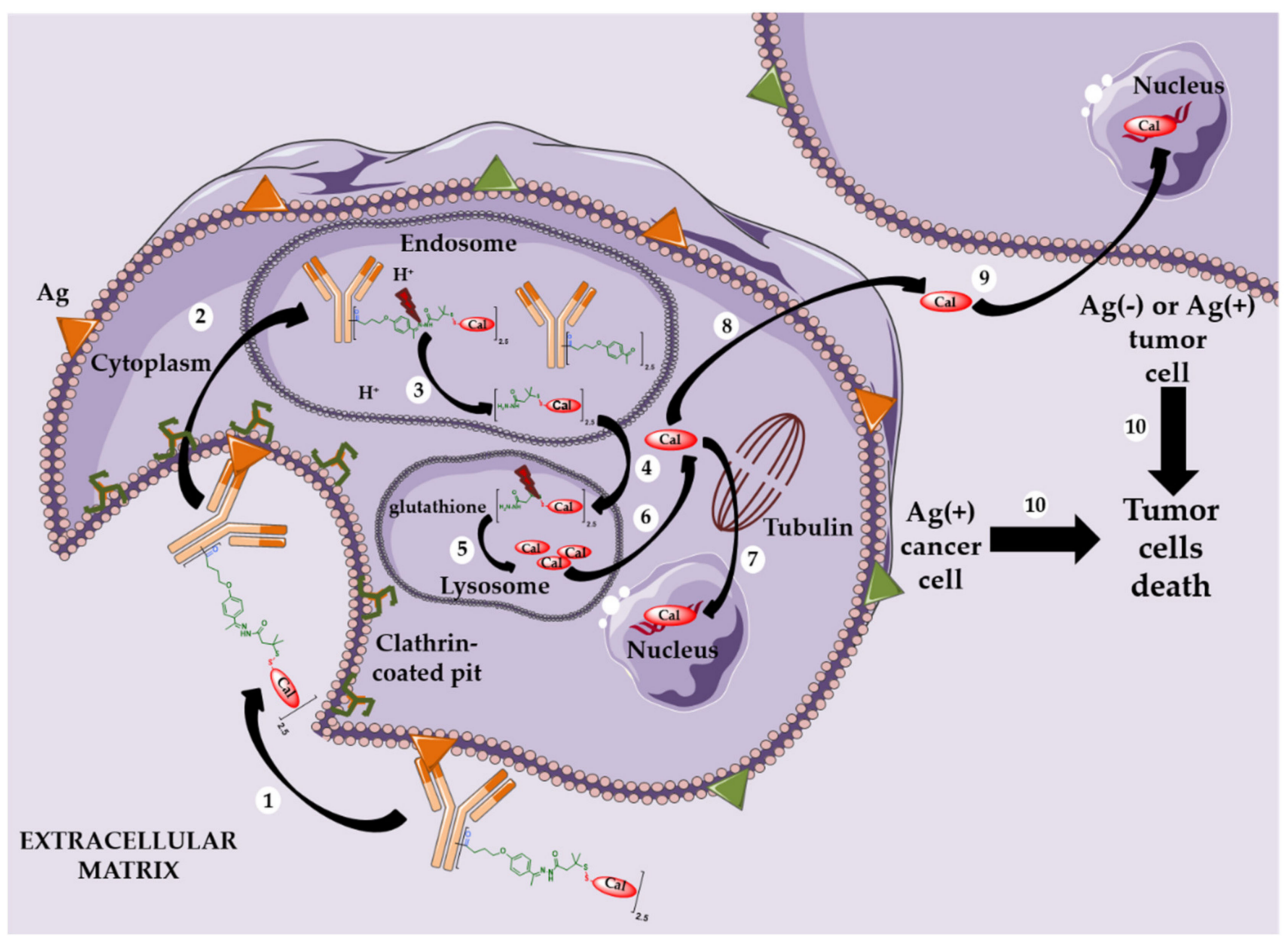
Mylotarg^®^ or Besponsa^®^ mechanism of action: (1) binding to the specific antigen (Ag), followed by internalization of the ADC-Ag complex through a clathrin-dependent mechanism; (2) transfer to endosomes; (3) the acid-sensitive hydrazone of the linker is cleaved in early endosome and lysosome; (4) transfer of pro-calicheamycin into the lysosome; (5) cleavage of the disulfide bridge to give calicheamycin then (6) transfer of calicheamycin into the cytoplasm and (7) transfer of calicheamycin into the nucleus. (8) Alternatively to 7, transfer of calicheamycin into the cytoplasm then (9) diffusion of calicheamycin into neighboring cancer cells to obtain a cytotoxic bystander effect and (10) all the previous steps lead to cell death.

Mylotarg^®^ (Gemtuzumab ozogamicin) was approved by the FDA in 2000 for acute myeloid leukemia (AML) [11,12]. Mylotarg^®^ results from the conjugation of calicheamycin, a powerful DNA-cleaving agent [13] with low nanomolar activity, onto gemtuzumab, a mutated anti-CD33 IgG4, via a cleavable linker including an hydrazone bond (Figure 1). This ADC, with an average DAR of 1.5, is a complex mixture with around 50% of unconjugated mAb [14]. After ADC internalization, the hydrazone bond can be hydrolyzed in the endosomal acidic environment to release a precursor of calicheamycin, which then undergoes a reduction by glutathione to the free active calicheamycin. The latter binds to the DNA minor groove and undergoes a Bergman cyclization, which generates a highly reactive diradical causing sequence-selective double-strand cuts (Scheme 1).

Theoretically, hydrazones should be stable in blood circulation at physiological pH and undergo selective hydrolysis after internalization in more acidic conditions (respectively pH 5.0–6.5 in endosomes and pH 4.5–5.0 in lysosomes).

However, the Mylotarg^®^ linker exhibits a certain instability, leading to premature release of calicheamycin in plasma circulation [15], explaining its highly toxic profile and its subsequent voluntary withdrawal by Pfizer in 2010.

Mylotarg^®^ benefited from the knowledge accumulated in the clinic in recent years to be reapproved by the FDA in 2017, used at lower doses, with a modified administration schedule and for a different patient population.

A similar linker was developed and used to graft calicheamycin onto inotuzumab, a mutated anti-CD22 IgG4, leading to Besponsa^®^ (inotuzumab ozogamicin, Figure 1), approved by the FDA in 2017 against acute lymphoblastic leukemia (ALL) [16].

### 2.2. Kadcyla^®^ and the Notion of Second Generation Non-Cleavable Linkers

Given these findings, alternative strategies for the design of linkers were necessary to continue the development of ADCs. Thus, Immunogen teams have focused on the use of linkers for the conjugation of maytansine derivatives and incorporating a delivery system using a glutathione-sensitive disulfide bond [17]. These innovative chemically labile linkers were intended to allow controlled release in the presence of glutathione (GSH), the cytoplasmic concentration of which in cancer cells is approximately 1000 times higher than in plasma.

In addition, the careful positioning of two methyl groups neighboring the disulfide bond enabled control of the release kinetics [18]. Thus, the high concentration of reducing molecules in the tumor should have guaranteed a selective release of the payload in the tumor environment but not in the circulation. This type of linker has not yet led to an ADC approved on the market (see Section 5.6).

**Scheme 2:**
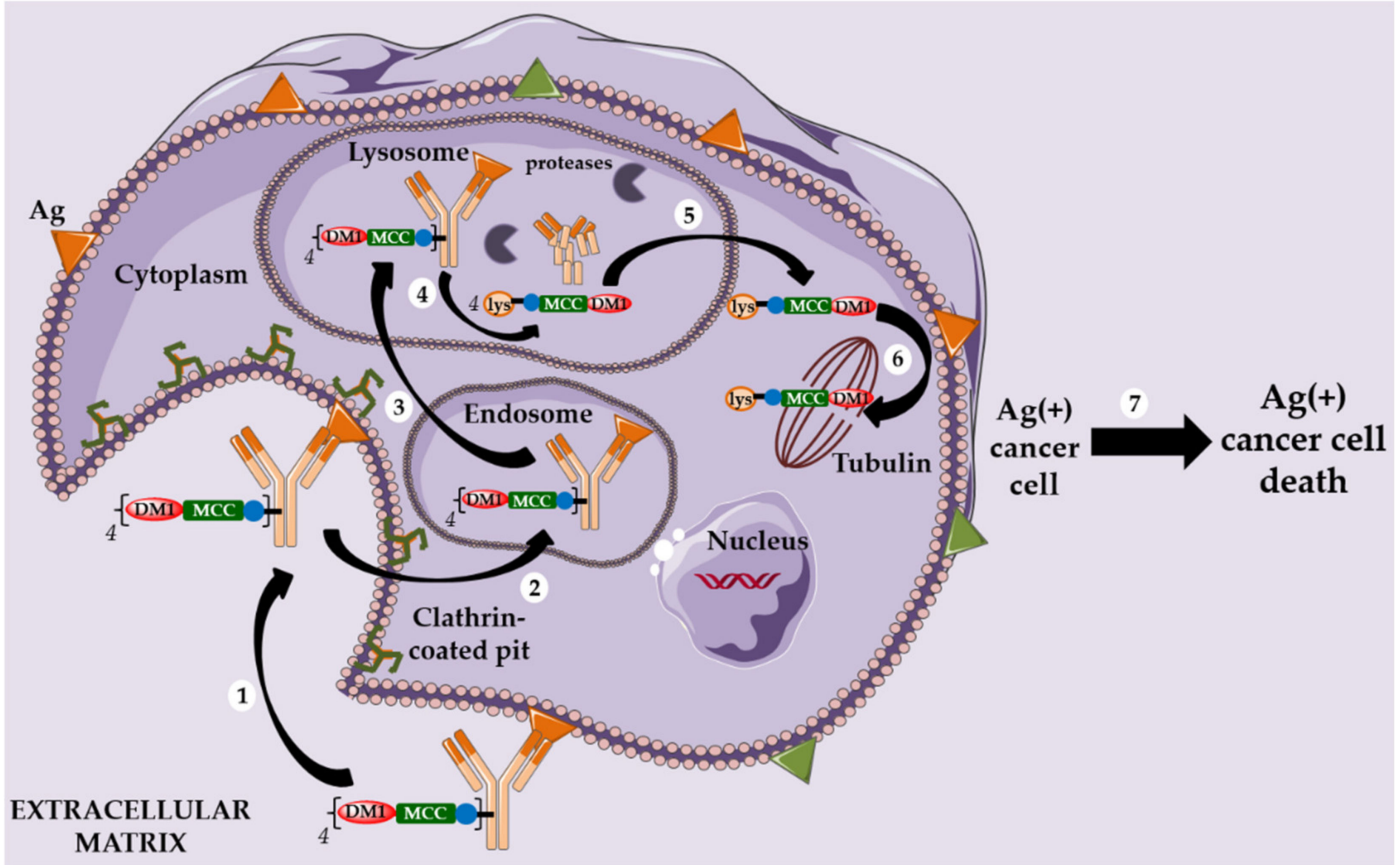
Kadcyla^®^ mechanism of action: (1) binding on its specific HER2 Ag, followed by internalization of the T-DM1-HER2 complex by a clathrin-dependent mechanism; (2) transfer to the endosome then (3) to the lysosome; (4) complete trastuzumab digestion to release the active metabolite, LYS-MCC-DM1 and (5) transfer into the cytoplasm. (6) due to its charge at the physiological pH, the active metabolite is unable to cross the cell membrane, therefore it does not elicit any bystander killing effect, and transfer to tubulin. (7) All the preceding steps lead to HER2-positive cancer cell death.

However, a serendipitous finding allowed Immunogen to identify an unexpectedly effective ADC. The conjugation of DM1 onto the lysine residues of anti-HER2 IgG1 trastuzumab via a non-cleavable heterobifunctional thioether linker containing an *N*-hydroxysuccinimide ester (succinimidyl-4-(*N*-maleimidomethyl) cyclohexane-1-carboxylate or SMCC) led to Kadcyla^®^ (T-DM1 or Ado-trastuzumab emtansine, Figure 1), which was approved by the FDA in 2013 [19]. This new ADC was observed in vitro to be very potent in a HER2-positive breast cancer model, the original structure being active only after internalization and complete enzymatic digestion of the ADC in the lysosome, to obtain the active metabolite lysine-MCC-DM1 (Scheme 2).

Following this discovery, several observations can be highlighted: (i) the metabolite lysine-MCC-DM1 retains the cytotoxic potential of free DM1, allowing the corresponding ADC to reach an in vitro activity in the picomolar range; (ii) this ADC exhibits no bystander killing effect due to the charged nature of its active metabolite at physiological pH; (iii) an ADC with a non cleavable linker can target only Ag-positive cells and (iv) this ADC has a limited toxicity on normal tissues and is more stable during circulation than an ADC with a cleavable linker.

### 2.3. Adcetris^®^, Polivy^®^ and the Second-Generation Cleavable Linker

In parallel, Seattle Genetics has designed its own linker technology allowing the bioconjugation of dolastatin derivatives (such as monomethyl auristatin E or MMAE) onto the cysteine residues of an anti-CD30 IgG1 to produce Adcetris^®^ (SGN-35 or brentuximab vedotin, Figure 1) [20,21,22]. After mild and partial reduction of the interchain disulfide bridges, the anti-CD30 mAb (cAC10) was bioconjugated to a cleavable heterobifunctional maleimide linker. This maleimidocaproyl-valine-citrulline-*p*-aminobenzyloxycarbonyl linker includes a valine-citrulline peptide trigger (ValCit) sensitive to lysosomal cathepsin B and a para-aminobenzyl alcohol (PAB) as a self-immolative spacer (SIS) allowing the release of MMAE after internalization in CD30-positive tumor cells.

**Scheme 3:**
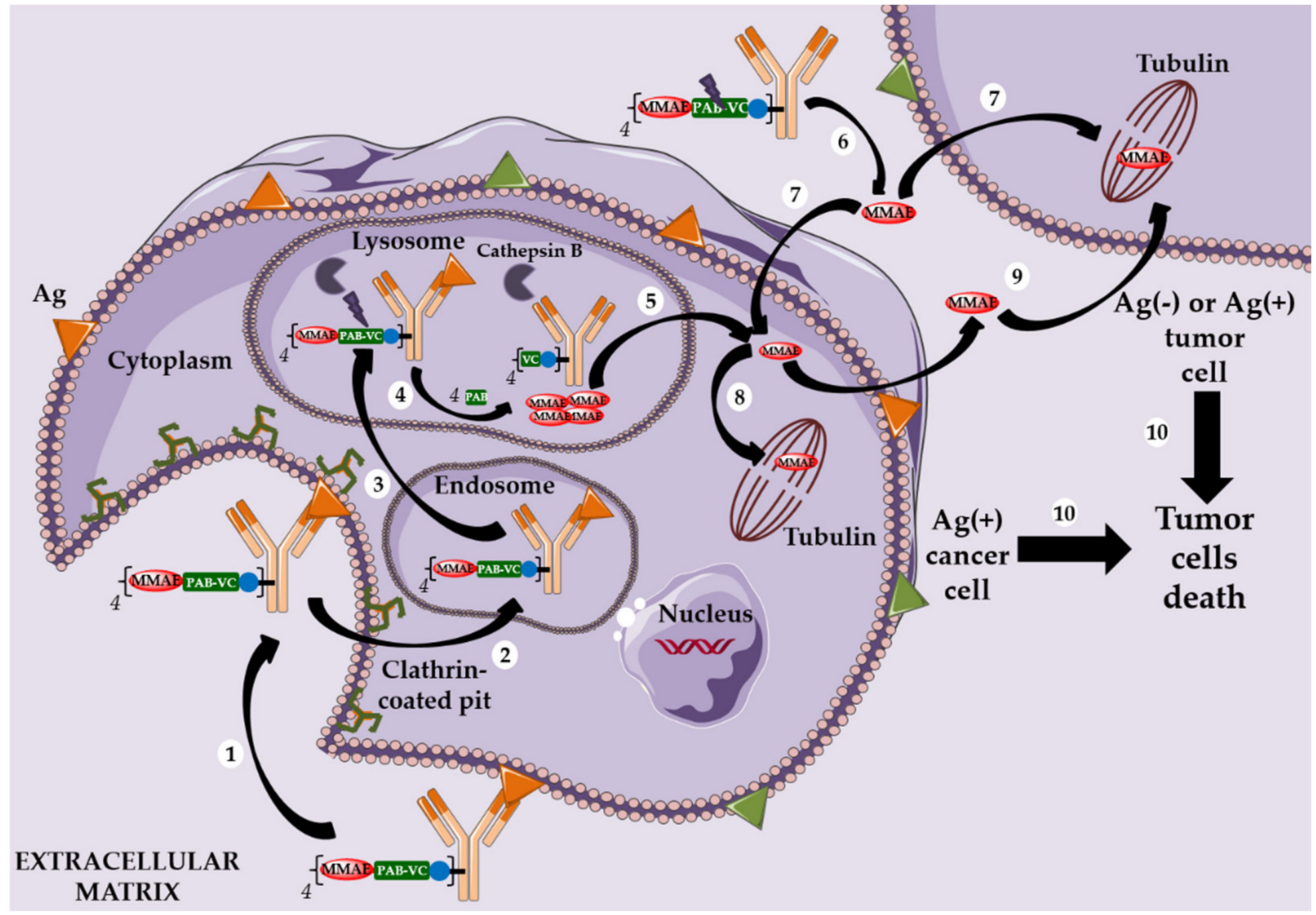
Adcetris^®^, Polivy^®^ or Padcev^®^ mechanism of action: (1) binding to a specific Ag, followed by internalization of the ADC-Ag complex according to a clathrin-dependent mechanism; (2) transfer to the endosomes then (3) to the lysosomes; (4) linker cleavage in the lysosomes takes place between the peptide sequence (ValCit) and the self-immolative spacer (PAB); (5) transfer of MMAE into the cytoplasm; (6) MMAE can also be released before internalization, then (7) enter the targeted cell (or a nearby tumor cell) and (8) intracellular or extracellular MMAE release is followed by tubulin targeting. In parallel to 8, (9) diffusion of another MMAE in neighboring tumor cells not targeted by the ADC to obtain a bystander killing effect and (10) all the previous steps lead to tumor cell death.

Adcetris^®^ was approved by the FDA in 2011 and for the treatment of anaplastic large cell lymphoma and Hodgkin’s lymphoma. After CD30-dependent ADC internalization, followed by degradation of the cleavable linker, the released MMAE can destroy the targeted cell and diffuse across the plasma membrane to reach and kill the neighboring cancer cells. This phenomenon is called the bystander killing effect [23,24] and allows the released MMAE to kill CD30-positive and CD30-negative tumor cells (Scheme 3) [25]. By corollary, the bystander killing effect explains the particular in vivo efficacy of Adcetris^®^ in patients treated for heterogeneous lymphomas. Recently, Neri and his team demonstrated that the linker ValCit-PAB used in this ADC can also be cleaved before internalization [26], helping to dispel the dogma stipulating that an ADC must target an internalizing antigen [27] to be relevant (see Section 5.4).

Similarly, this second generation linker (mc-VC-PAB, Figure 1) was used to conjugate MMAE onto polatuzumab, an anti-CD79b IgG1, leading to Polivy^®^ (polatuzumab vedotin-piiq, Figure 1), approved by the FDA in June 2019 in combination with bendamustine-based chemotherapy and rituximab, to treat adult patients with diffuse large B-cell lymphoma (DLBCL) [28,29].

We also found the same linker-drug (Figure 1) in enfortumab vedotin-ejfv, an ADC resulting from a stochastic bioconjugation on the cysteine residues of an IgG1 targeting Nectin 4, developed by Seattle Genetics, currently in a pivotal phase 2 clinical study [30]. On 16 July 2019, a BLA (Biologics License Application) for this ADC was submitted to the FDA for possible accelerated marketing for patients suffering from metastatic urothelial cancer and previously treated with anti-PD1/PDL1 antibodies. Enfortumab vedotin-ejfv was finally approved by the FDA as Padcev^®^ in late December 2019.

Nevertheless, the molecular architecture of Adcetris^®^, Padcev^®^ or Polivy^®^ has two limitations. The first is related to the instability of the maleimide used for bioconjugation, which is capable of undergoing a retro-Michael reaction in plasma, causing a partial deconjugation and then a slow transfer to albumin possibly correlated with off-target toxicity [31]. The second concerns the “ValCit” trigger described for Adcetris^®^ as a substrate of carboxylesterase 1C during plasma circulation and of other proteases secreted by neutrophils (for example, elastase), which could partly explain the hematological undesirable effects related to Adcetris^®^ (for example, neutropenia) [32,33,34].

## 3. Toxicity

The use of highly toxic ADCs has changed the paradigm of immunotherapy directed against tumor antigens, with limited side effects regarding the level of expression of the targeted antigen. Indeed, the two most used antibodies, rituximab directed against CD20 and trastuzumab directed against Her2, both have a toxicity profile allowing them to be combined with conventional chemotherapy agents without redundant toxicity. In the case of ADCs, the situation is different, because certain side effects are similar to some of those induced by conventional cytotoxic agents used in chemotherapy, while others are specific to the conjugates themselves. The occurrence of these side effects, sometimes severe, is partly explained by the uncontrolled release of the highly cytotoxic drug in the circulation, responsible for off-target toxicity. In addition, the IgG1 isotype of some of these ADCs can engage the Fc-gamma receptors (FcγR), which can trigger a target-independent, FcγR–dependent internalization in FcγR-positive cells resulting in toxic effects on these untargeted healthy cells. The development of these agents, either as monotherapy or in combination, has therefore proven to be complex, as evidenced by gemtuzumab ozogamicin.

### 3.1. Gemtuzumab Ozogamicin or the Roller Coaster of the First ADC Approved in Humans

Gemtuzumab ozogamicin (Mylotarg^®^) was approved in 2000 by the FDA for the treatment of certain patients with acute myeloblastic leukemia (AML). In 2001 the FDA issued an alert due to the observation of cases of veno-occlusive disease. In 2004 a randomized study comparing a conventional treatment with an arm combining Mylotarg^®^ was stopped prematurely due to an increased mortality rate in the latter. In 2010 Mylotarg^®^ was withdrawn from most markets, except in Japan. However, study 0701 by the Acute Leukemia French Association (ALFA) showed that the fractionation of the administration into three doses improved both survival without events and overall survival without significant additional toxicity, in particular on the hepatic level [35].

The case of Mylotarg^®^ is interesting for many reasons. Due to the limited analytical methods available in the first clinical studies, Mylotarg^®^ was only later identified as a heterogeneous product in terms of DAR. While the theoretical DAR was around 2.5, more than 50% of the antibodies contained in the pharmaceutical preparations were not substituted, while others had a DAR of 4 or 5. The development difficulties of Mylotarg^®^ also show the difficulty of identifying a dose regimen to obtain a satisfactory therapeutic index, and the need not to abandon the development of an ADC after exploring only a single dose regimen.

### 3.2. Brentuximab Vedotin for the Treatment of Hodgkin’s Disease

Brentuximab vedotin (Adcetris^®^), directed against CD30, was approved for the treatment of certain forms of lymphoproliferative syndromes expressing CD30 including Hodgkin’s disease. The cytotoxic drug of this ADC is the vedotin (monomethyl auristatin E, MMAE), a powerful antitubulin agent. This ADC has a strong antitumor activity in monotherapy, in anaplastic large cell lymphomas (ALCL) and refractory Hodgkin’s diseases (*NCT00848926*) [36,37].

However, Adcetris^®^, as monotherapy, has been associated with potential severe peripheral neuropathies, neutropenia and thrombocytopenia, classic side effects of antitubulin agents. On the other hand, rare but serious cases of progressive multifocal leukoencephalopathies were observed [38].

Additionally the combination of Adcetris^®^ with bleomycin, a commonly used agent for the treatment of Hodgkin’s disease, was found to lead to unacceptable pulmonary toxicity, precluding this combination [37].

### 3.3. The Classic or Unexpected Toxicities of ADC

Certain toxicities observed with immunoconjugates are expected since the conjugates target either tubulin and the mitotic spindle (auristatins and maytansinoids) or DNA (calicheamycin and PBD). These include bone marrow toxicity up to grade 4, sensitive neurological and vegetative toxicities.

On the other hand, several side effects, which are not observed with standard cytotoxic agents, have been reported. These include ocular toxicities such as keratitis or corneal deposits with ADCs containing MMAF or DM4, which may constitute limiting toxicity for these compounds [32]. Kadcyla^®^ has been shown to increase the risk of radionecrosis [39]. A better understanding and management of these unexpected toxicities will be essential for optimal use of these agents.

## 4. Mechanisms of Resistance to ADCs

The mechanism of action of ADCs at the level of the tumor targeted cell comprises of several stages: binding to the antigen, internalization, release of the conjugate (mainly in the lysosome), release of the conjugate into the cytoplasm and then binding to the molecular target of the conjugate inducing cell death by apoptosis. Each of these steps can be involved in resistance as suggested by several preclinical works on cell lines or in animal models: (i) downregulation of the targeted antigen [40,41] and/or defects in binding, internalization, trafficking or recycling of the antibody, (ii) defective lysosomal degradation of the ADC or reduced expression of lysosomal transporters such as SLC46A3 [42], leading to lower release of the payload in the cytosol, (iii) alterations of tubulin or microtubule dynamics modulators [43] or (iv) reduced drug retention within the cell by upregulation of multidrug resistance transporters like MDR1 [44,45].

The clinical relevance of these various potential resistance mechanisms remains to be demonstrated. Indeed, it is complex to have access to tumor samples immediately before the initiation of treatment with ADC and then during the relapse following such treatments. Finally, in the context of therapeutic combinations, it can be complex to discern the mechanisms of resistance to ADCs from those of the other administered compounds. Despite this, the observations made on preclinical models raise interesting avenues for the analysis of resistance to ADCs in humans.

## 5. New Strategies in Development: Third Generation ADC

### 5.1. Limits of Current Approaches

Many ADCs, in clinical development or in clinical use, are based on a complete internalizing IgG format, targeting an Ag with an extremely high overexpression rate, and are conjugated to tubulin polymerization inhibitors using stochastic bioconjugation techniques [1]. In addition, cleavable linkers are known to be unstable during plasma circulation, while hydrophobic linkers are associated with a higher aggregation propensity. However, ADCs based on a complete IgG are associated with tumor penetration issues in stroma rich tumors [46,47] and are recycled by the neonatal Fc receptor (FcRn) leading to an undesirable distribution in the endothelium and the liver, responsible for undesirable side effects. Moreover, after internalization, ADCs effectiveness is based on favorable intracellular trafficking to reach the lysosome, where their degradation will allow controlled drug release. However, this strategy has several limiting factors. Firstly, an ADCs internalization capacity is intimately correlated with the high expression of surface Ag (for example, CD30 and HER2) [48], which explains why these ADCs using conventional tubulin polymerization inhibitors (for example, auristatins and maytansinoids) do not show cytotoxic activity on cells with low antigenic expression. Very powerful drugs (for example, pyrrolobenzodiazepine (PBD) dimers) have been developed to overcome these limitations, but the corresponding ADCs have a limited therapeutic index, particularly in solid tumors. Secondly, internalizing ADCs, including Kadcyla^®^, induce tumor resistance by several mechanisms. In fact, disturbances concerning internalization, trafficking or recycling of mAb, Ag shedding and defective lysosomal degradation of ADCs lead to a reduced drug release into the cytosol, thus compromising ADCs efficacy [40,41,49]. There is therefore today an important need to develop new technologies concerning bioconjugation techniques (leading to homogeneous ADCs), the vector format (antibodies or fragments), the linker (release mechanism) or the drug (new mechanism of action). Each strategy will be discussed as a potential alternative to the heterogeneous internalizing ADCs used until 2019 in clinical practice, to widen the fields of ADCs application [2] and to lead to the recent approval of two third-generation ADCs.

### 5.2. Site-Specific ADCs

Despite their growing success, until 2019, each approved ADC on the market was in the form of a heterogeneous mixture (variable numbers of cytotoxic drugs on the mAb, in various places), leading to analytical issues [3] during the manufacturing process. Indeed, DAR is not controlled, and this complex mixture significantly influences ADC pharmacokinetics-pharmacodynamics profiles (PK-PD): the naked antibody could be a competitive inhibitor, conjugates with weak DAR have a poor efficacy and those with high DAR are rapidly eliminated in plasma, compromising the ADC therapeutic window. In order to broaden the therapeutic index of ADCs, regiospecific bioconjugation methodologies have been widely developed since 2008 [6,50,51]. These site-specific methodologies can be divided into three categories: (i) bioconjugation onto natural or non-natural amino acids, (ii) bioconjugation using enzymes or (iii) linker-based bioconjugation.

**Figure 2:**
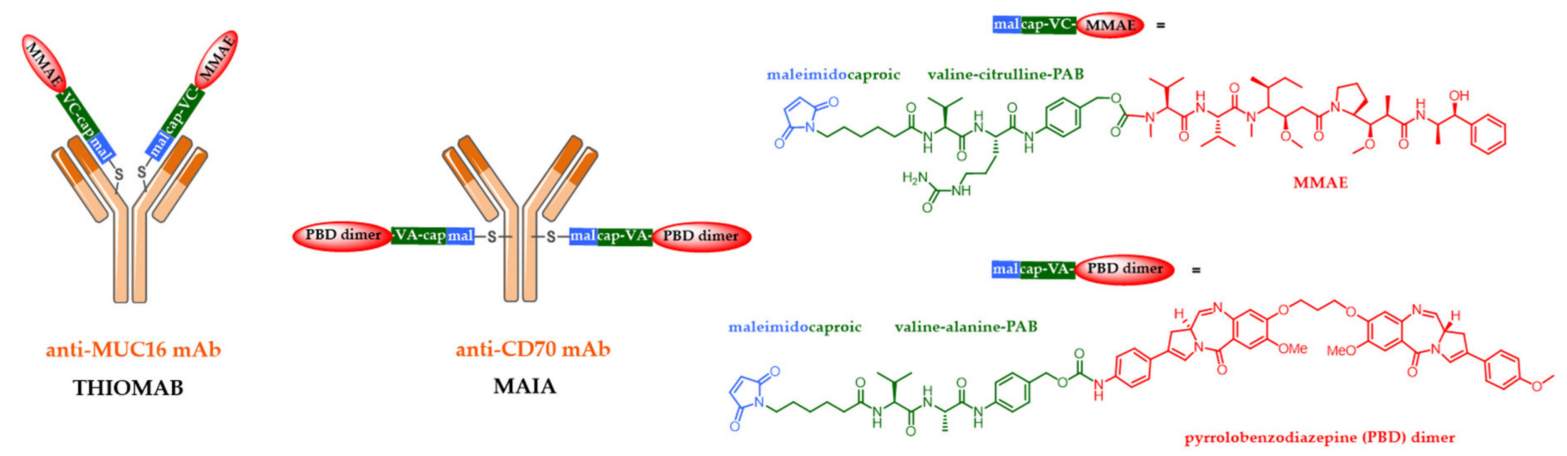
Homogeneous ADCs with a drug-to-antibody ratio (DAR) 2 generated through antibody engineering and site-specific bioconjugation.

A first approach consisted of introducing specific amino acids by antibody engineering. Junutula and his colleagues at Genentech were the pioneers in 2008 by demonstrating that the controlled site-specific bioconjugation of a cytotoxic onto a mAb improved ADC therapeutic index [52]. They developed an ADC, through the site-specific bioconjugation of the Adcetris^®^ linker at position 265 onto a mAb targeting the ovarian cancer antigen MUC16. For that purpose, they introduced two cysteines into the amino acid sequence of the mAb, which positions were chosen to keep IgG folding and Ag binding (Figure 2). The resulting TDC (ADC Thiomab) was compared with an ADC generated using a conventional stochastic bioconjugation method (on cysteines from reduced interchain disulfide bridges). Both ADCs were effective in mouse xenograft models, but TDC was tolerated at higher doses than its ADC counterpart in rats and cynomolgus monkeys and exhibited lower systemic toxicity in vivo. Inspired by this strategy, Seattle Genetics and Spirogen have developed a similar technology, called MAIA, for the bioconjugation of their PBD dimers, by introducing a serine-cysteine mutation at position 239 in the mAb pivotal region (Figure 2) [53,54].

**Figure 3:**
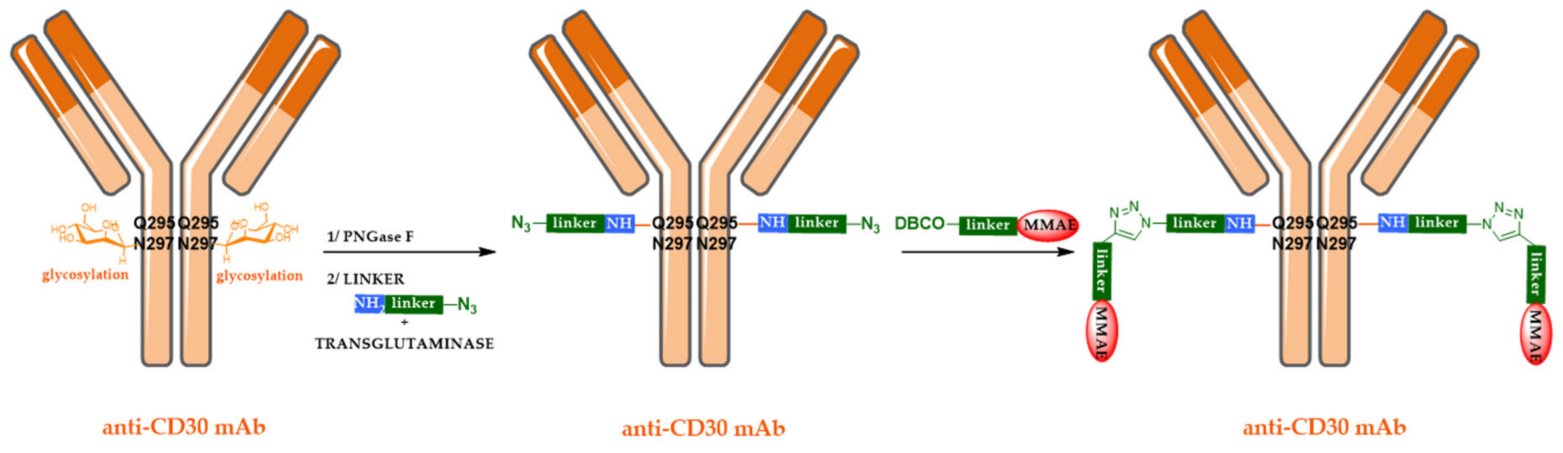
Homogeneous ADC generated by deglycosylation (position Q295), in the presence of PNGase F, followed by a bioconjugation mediated by transglutaminase (position 297). The azide immunoconjugate, in the presence of DBCO-linker-MMAE, allows the production of a homogeneous ADC with a DAR 2.

A second possibility is the use of enzyme-mediated regiospecific bioconjugation technologies. Transglutaminase and sortases can be used for this purpose [6,50,51]. For example, transglutaminase catalyzes the formation of amide bonds between the glutamine side chain of a native mAb (without modification by bioengineering) and a molecule containing a primary amine [55]. Thus, Schibli et al. (Innate Pharma) have developed a three-step methodology using transglutaminase to build an ADC [55]. The mAb is firstly deglycosylated by the PNGase F enzyme on asparagine 297. Next, an amino and nitrogen heterobifunctional linker is bioconjugated onto the mAb at glutamine 295 in the presence of microbial transglutaminase. Finally, MMAE is grafted via a copper-free Huisgen cycloaddition (SPAAC) between the azide conjugate and a linker carrying dibenzylcyclooctyne (DBCO) and MMAE, in order to generate an ADC with a DAR of 2 (Figure 3).

**Figure 4:**
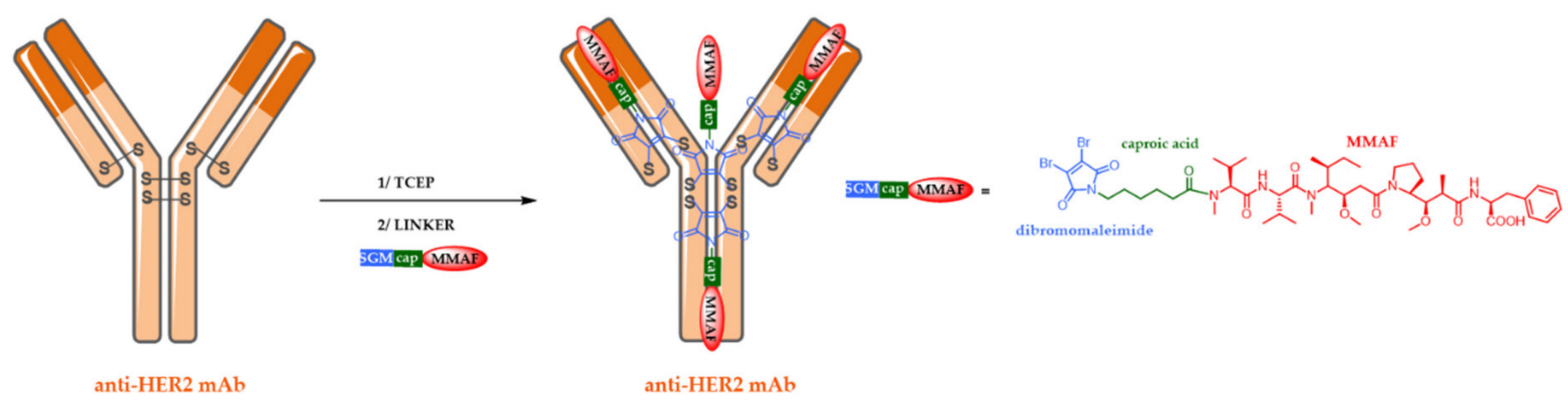
Homogeneous ADC with a DAR 4 obtained by complete reduction (TCEP) of all 4 interchain disulfide bridges followed by a site-specific bioconjugation reaction via a dibromomaleimide linker (DBM).

Finally, regiospecific ADCs can also be generated from native mAbs. In this strategy, heterobifunctional linkers comprising of innovative dibromomaleimide (DBM) [56,57] or dithiophenylmaleimide (DSPh) [58,59] can generate more homogeneous and more stable ADCs by regiospecific bioconjugation, with a DAR of 4 (in the case of IgG1). These two bioconjugation systems offer the advantage of better regiospecificity than classical cysteine conjugation compared to classical maleimide, while limiting the formation of species with low and high DAR. They also improve the structural stability of antibodies after bioconjugation [60]. In particular, DSPh [58] has excellent synthetic accessibility and good hydrolysis stability, while DBM (Figure 4), in combination with the cytotoxic monomethyl auristatin F (MMAF, an analogue of MMAE), have presented improved pharmacokinetic properties and therapeutic index compared to conventional heterogeneous ADCs obtained with a first generation maleimide linker via a stochastic conjugation method.

### 5.3. Alternative ADC Formats

Despite their efficacy, most ADCs targeting solid tumors have not progressed beyond phase 2 clinical trials, which suggests that there are additional parameters that need to be optimized in order to reach the market [61,62]. The number of ADCs for solid tumor treatment is limited, and a number of these conjugates have been discontinued due to insufficient activity at repeated doses of DMT. This can be explained by the fact that almost all ADCs are based on a complete immunoglobulin G (IgG) format. Consequently, their effectiveness is limited by their size (150 kDa), associated with poor penetration and absorption in the tumor [63]. In addition to the size of the IgG, it is now considered that the Fc part of an IgG can be unnecessary or even undesirable for ADCs efficacy [64], although some ADCs (e.g., Kadcyla^®^) retain the ability of their mAb to exhibit antibody-dependent cell-mediated cytotoxicity (ADCC) and antibody-dependent cellular phagocytosis (ADCP). Indeed, ADCs long half-life induced by the FcRn [65] increases exposure to healthy tissues, while FcγR cross-react with endothelial cells and the immune system, these two phenomena being associated with off-target toxicity [66,67].

Smaller conjugate formats [64,68,69] have been explored to remedy these drawbacks, in particular peptides [70], single domain antibody fragments (sdAb or VHH) [71], single chain variable fragments (scFv) [72], antigen binding fragments (Fab) [73] or small immunoproteins (SIP) in the form of scFv dimerized using a CHε4 domain [74,75]. Surprisingly, only a few examples of drugs effectively vectorized with these new antibody formats have been published: Janda’s team has described several scFv-duocarmycin conjugates with a DAR of 1 or 2, targeting laminin-binding integrin α_3_β_1_, with an EC_50_ between 2.7 and 180.8 nM on pancreatic carcinoma cells SW1990 [76]. The Godwin team described a site-specific Fab-MMAE conjugate, with a site-specific DAR of 1, targeting HER2 and having an EC_50_ of 0.2 nM on SK-BR-3 breast cancer cells [73]. More recently, the Spidel team has generated two optimized scFv-auristatin F conjugates with a DAR of 2, targeting CA9, exhibiting similar efficacy with an EC_50_ of 0.57 and 0.81 nM on colon cancer cells HT116- CA9 [77].

**Figure 5:**
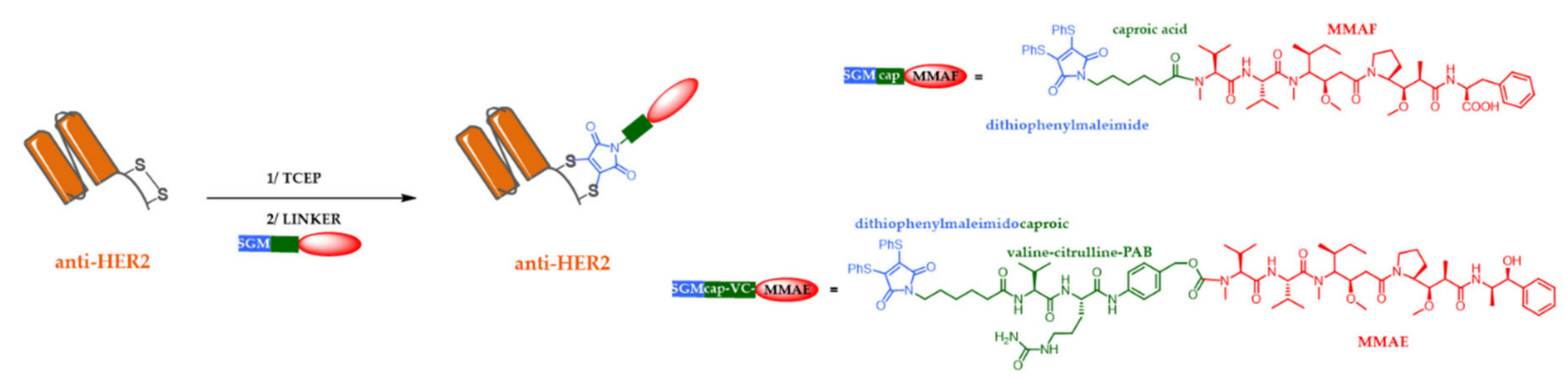
Homogeneous SDC (scFv-drug conjugate) with a DAR 1 obtained by reduction (TCEP) of the C-terminal intrachain disulfide bridge followed by a site-specific bioconjugation reaction via a dithiophenylmaleimide linker (DSPh).

As part of this strategy, more recently, the site-specific conjugation of an auristatin derivative to an anti-HER2 scFv (derived from trastuzumab) generated two new scFv-drug conjugates (SDCs, Figure 5) [78]. Two cysteines have been judiciously incorporated near the hexahistidine tag (at the C-terminal position) in order to allow controlled bioconjugation of a heterobifunctional linker comprising of a second generation maleimide, cleavable (for MMAE) or non-cleavable (for MMAF) [2]. The two SDCs retain their affinity for HER2 in comparison to the native scFv and are capable of effectively killing SK-BR-3 HER2-positive cells in vitro at subnanomolar concentrations (EC_50_ of 0.68 nM and 0.32 nM, respectively). No effect was observed on HER2-negative MCF-7 cells. Although SDCs with a DAR of 1 are not yet as powerful as the corresponding ADCs with a DAR of 4 [79], this work represents the first step towards the design of more efficient small conjugates with higher DARs, opening up the path to further in vivo or even (pre)clinical studies in order to assess their promising potential against solid tumors [80].

**Figure 6:**
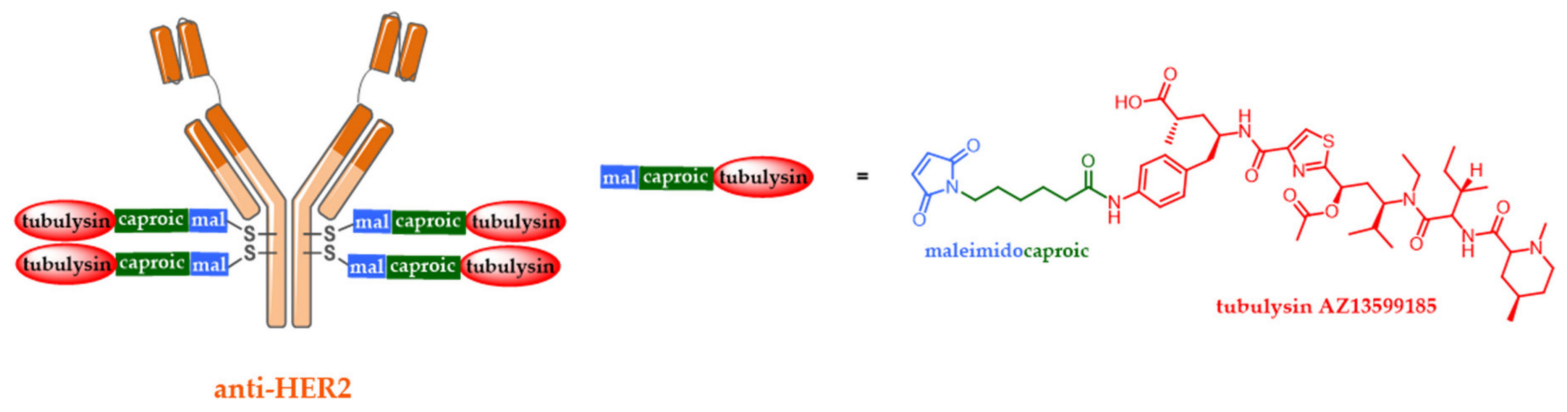
Homogeneous ADC with a DAR 4 comprising a biparatopic anti-HER2 antibody conjugated to a tubulysin derivative via a classical maleimidocaproic linker.

Current ADCs targeting HER2 are ineffective in removing cancer cells expressing relatively low HER2 levels of expression. As a result, only about 20% of breast cancer patients are eligible for targeted HER2 therapies. In addition, the intratumoral heterogeneity of HER2 expression is ultimately responsible for the relapse of patients who initially responded to treatment. In order to obtain potent antitumor activity in cancer cells with a wider range of HER2 expression, the biparatopic ADC IMMU4276 targeting HER2 was developed by MedImmune (Figure 6) [81]. To build this impressive molecular architecture, a bivalent biparatopic mAb was first designed to target two distinct HER2 epitopes (epitopes individually targeted by trastuzumab and pertuzumab), inducing HER2 receptor dimerization, promoting robust internalization, as well improved trafficking and lysosomal degradation. Two heavy chain cysteine residues (S239C and S442C) were introduced by antibody engineering, to produce a biparatopic ADC of DAR 4, via the regiospecific bioconjugation of a maleimidocaproyl linker carrying a new inhibitor of microtubules based on tubulysine AZ13599185. AZ13599185 is a very powerful cytotoxic agent (IC_50_ in the picomolar range) and has a very low affinity for MDRs (multidrug resistance proteins: transmembrane proteins ensuring the efflux of xenobiotics out of cells).

Therefore, this biparatopic ADC demonstrated superior antitumor activity in comparison to Kadcyla^®^ in various tumor models representing various subpopulations of patients. In addition, two combined mutations (L234F and S239C) reduced binding to FcγR in order to minimize ADC aspecific internalization (independent of HER2) in normal tissues mediated by FcγR, thereby reducing the occurrence of side effects such as thrombocytopenia [82]. Unfortunately, this ADC was also stopped in the clinic in 2018, due to high hepatotoxicity.

### 5.4. New Targets and Associated Release Systems

Targeting internalizing antigens on the surface of cancer cells can be extremely difficult in solid tumors rich in intercellular stroma. Thus, a new approach has been developed, consisting of tumor microenvironment (stroma or vasculature) targeting instead of cancer cells [83]. In this strategy, extracellular proteases and other components of the extracellular matrix (such as acidic medium or reducing glutathione) could be used for effective extracellular release of cytotoxics used in non-internalizing ADCs [2].

To this end, Neri and his collaborators have described immunoconjugates derived from a F8 mutant of a SIP targeting the non-internalizing extracellular domain (EDA) of fibronectin.

Fibronectin is a component of the tumor subendothelial extracellular matrix, easily accessible to immunoconjugates in comparison to the cancer cell surface [84].

**Figure 7:**
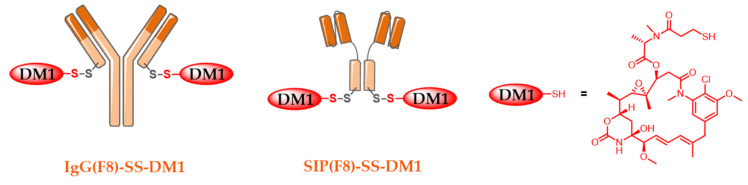
Immunoconjugates IgG(F8)-SS-DM1 and SIP(F8)-SS-DM1 with a DAR 2, and formula of the payload DM1.

The SIP format results from the fusion of a scFv fragment with the human IgE εCH4 domain [84,85]. SIP(F8) was also produced with two unpaired cysteine residues at the C-terminal position, allowing regiospecific bioconjugation of two DM1 molecules in order to produce the SIP(F8)-SS-DM1 conjugate with a DAR 2 (Figure 7) [74]. This was compared to its IgG(F8)-SS-DM1 counterpart, carrying an unpaired cysteine residue in the C-terminal position of each light chain (Figure 7). In these immunoconjugates, the disulfide bridge, formed after bioconjugation between the protein and the drug, is sensitive to extracellular tumor-derived glutathione, releasing unmodified DM1 without any residual linkers.

SIP(F8)-SS-DM1 has been shown to be a better candidate than its IgG(F8) -SS-DM1 analogue in therapeutic experiments. The two conjugates were compared in vivo in an F9-teratocarcinoma murine model at equal (equimolar) doses, in a group of five mice, with five injections at 5 mg/kg. The SIP conjugate led to the complete tumor remission in four out of five mice, while only limited tumor remission was observed for the IgG conjugate. The two conjugates have also been shown to present significant differences in terms of release kinetics of the cytotoxic DM1. Neri and his colleagues demonstrated that SIP(F8)-SS-DM1 preferentially accumulates in the subendothelial extracellular tumor matrix and rapidly generates high concentrations of released DM1, contributing to its higher efficiency. This conjugate has also been well tolerated at clinically relevant doses [75]. The advantage of non-internalizing ADCs is the circumvention of certain resistance mechanisms of internalizing immunoconjugates, while the regiospecific bioconjugation method once again allows control of DAR without the presence of DAR 0 species (native unconjugated protein).

### 5.5. New Cytotoxic Agents

In parallel, new cytotoxics have been developed to target cancer cells with low Ag expression or resistance to auristatins or maytansinoids.

**Figure 8:**
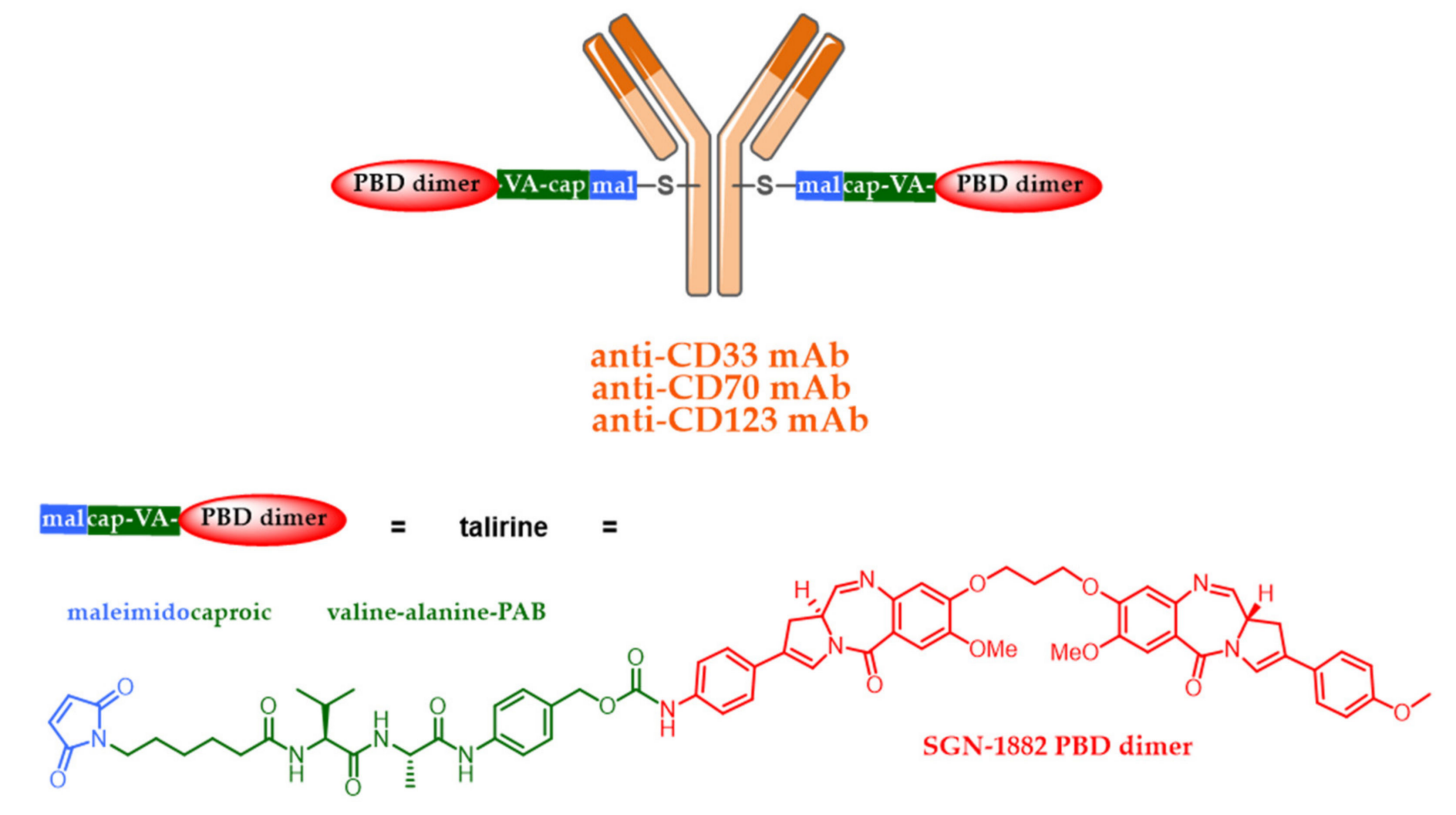
Homogeneous ADC with a DAR 2 carrying talirine including a dimeric derivative of pyrrolobenzodiazepine (PBD; SGN-1882).

To this end, PBD dimers have been developed. The molecular structure of these dimers contains two alkylating imine functionalities, capable of forming covalent bonds with DNA. Due to the nature of the DNA adducts and their greater stability, PBD dimers generally have significantly higher antitumor cytotoxicity than PBD monomers. They are approximately 50–100 times more effective than the conventional cytotoxics used in ADC (MMAE or DM1), exhibiting picomolar activity against many human tumor cell lines (IC_50_ = 2–7 pM) [86]. PBD dimers were introduced as ADC cytotoxics by Spirogen and Seattle Genetics in the late 2000s. This led to the development of SGN-CD33A (vadastuximab talirine) [53] and SGN-CD70A [54], two ADCs based on PBDs which unfortunately were recently stopped in phase III clinical trials. SGN-CD33A was associated with a higher rate of patient death due to fatal infection and possible liver toxicity, while SGN-CD70A was responsible of thrombocytopenia. Both ADCs contain the same cleavable linker drug called talirine; a maleimidocaproyl linker with a valine-alanine-PAB trigger sensitive to cathepsin B, capable of conjugating the PBD dimer SGN-1882 on two cysteine residues incorporated by antibody engineering in the hinge region of the mAb (mutation S239C), giving a controlled DAR of 2 (Figure 8). The high toxicity of these ADCs allows the targeting of less expressed Ag, such as CD33 or CD70. In addition, since PBD dimers are not substrates of MDR, they can also be used as ADC cytotoxics against tumors resistant to classical MMAE- or DM1-ADCs.

**Figure 9:**
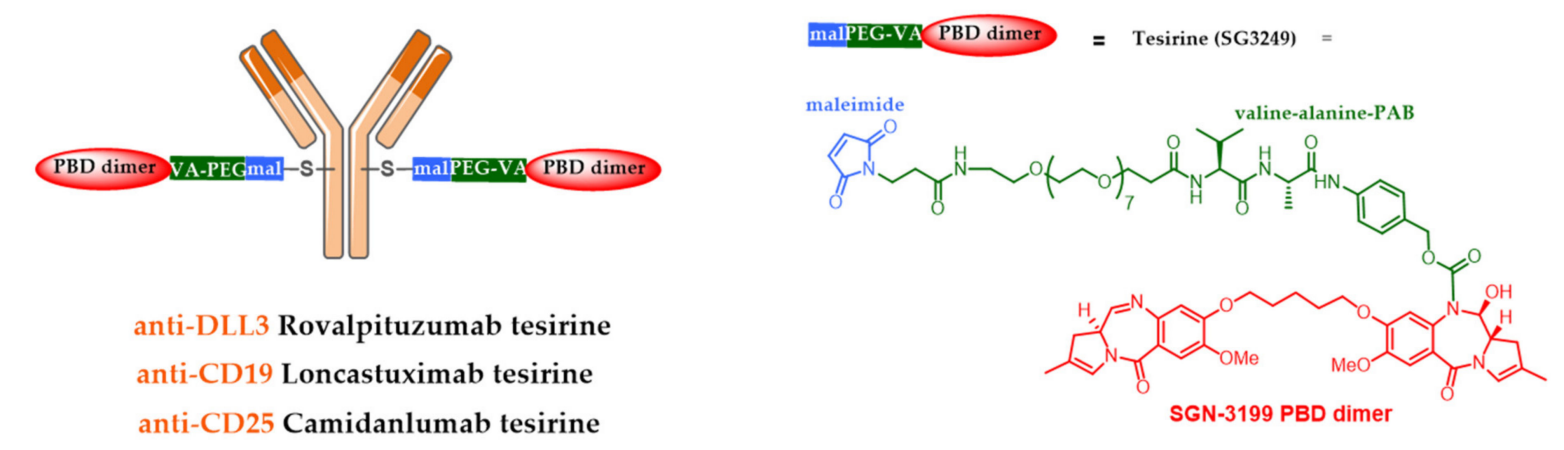
Homogeneous ADCs with a DAR 2 carrying tesirine including a dimeric derivative of PBD (SGN-3199).

Although ADCs using talirine were stopped during clinical trials, two ADCs using the counterpart tesirine are in an advanced phase of clinical study. Rovalpituzumab tesirine (Rova-T or SC16LD6.5) is an anti-DLL3 ADC developed by AbbVie (Stemcentrx) tested in phase III in small cell lung cancer [87,88]. While ADC Therapeutics developed loncastuximab tesirine (ADCT-402) [89] and camidanlumab tesirine (ADCT-301) [90], both tested in pivotal phase II, respectively against B cell acute lymphoblastic leukemia and Hodgkin lymphoma (Figure 9). Tesirine (SG3249) was designed to combine powerful antitumor activity with desirable physicochemical properties (e.g., favorable hydrophobia and improved bioconjugation). One of the reactive imines is capped with a valine-alanine linker cleavable by cathepsin B.

Similarly, in 2009, Ravi Chari and his colleagues at Immunogen presented indolinobenzodiazepines (IGN) [91,92] as extremely potent molecules (IC_50_ = 1–10 pM) against several cancer cell lines. Their use as cytotoxic payloads for ADCs has given rise to very potent ADCs against normal and multidrug-resistant cancer cell lines in vitro (IC_50_ = 5–20 pM). Even cell lines expressing low levels of Ag (7000 Ag per cell) have been effectively destroyed in vitro by an IGN-based ADC with an IC_50_ of 4 pM [83].

**Figure 10:**
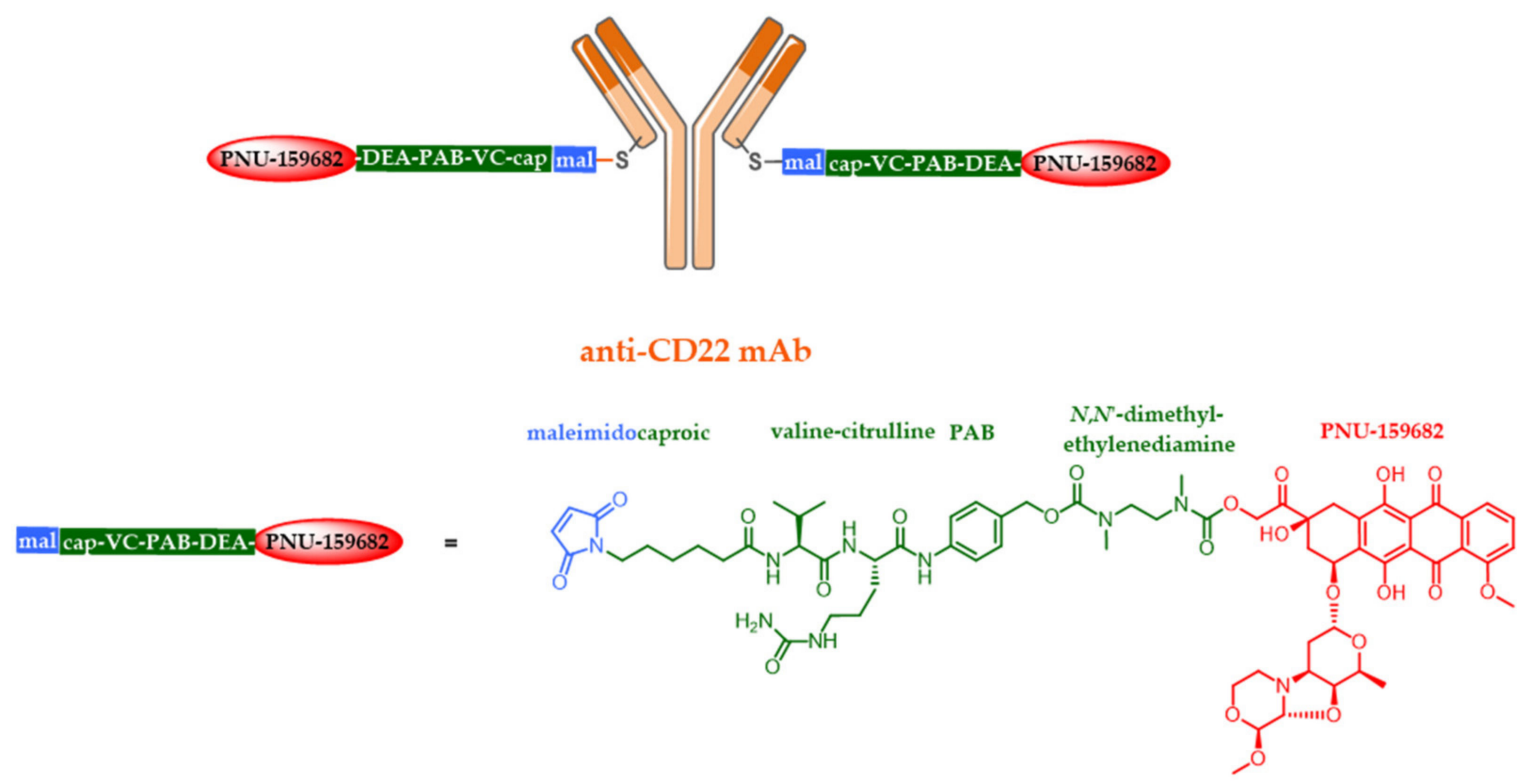
Homogeneous ADC carrying an anthracycline derivative (PNU-159682).

In parallel, Genentech recently developed an ADC (Figure 10), with a new anthracycline analogue named PNU-159682. Anthracyclines are one of the most widely used classes of chemotherapy and are very effective in the treatment of aggressive non-Hodgkin’s lymphoma (NHL). However, doxorubicin derivatives cannot be used as ADC cytotoxics due to a lack of activity, as we previously explained. On the other hand, the very powerful PNU-159682, with an IC_50_ of 20–100 pM, can be used in an ADC.

Genentech has developed a new anti-CD22-NMS249 ADC, using an MC-VC-PAB-DEA linker, with an elongated self-immolative spacer comprising of, in addition to the conventional PAB, an *N*,*N*′-dimethylethylenediamine (DEA; Figure 10) [44]. In vivo, this anti-CD22-NMS249 ADC was at least as effective as an anti-CD22-VC-MMAE ADC in xenograft tumor models but retained its effectiveness in a model based on cell lines resistant to the anti-CD22-VC-MMAE ADC. These results demonstrate the usefulness of anthracycline-based ADCs in the treatment of MMAE-resistant cancers [44].

**Figure 11:**
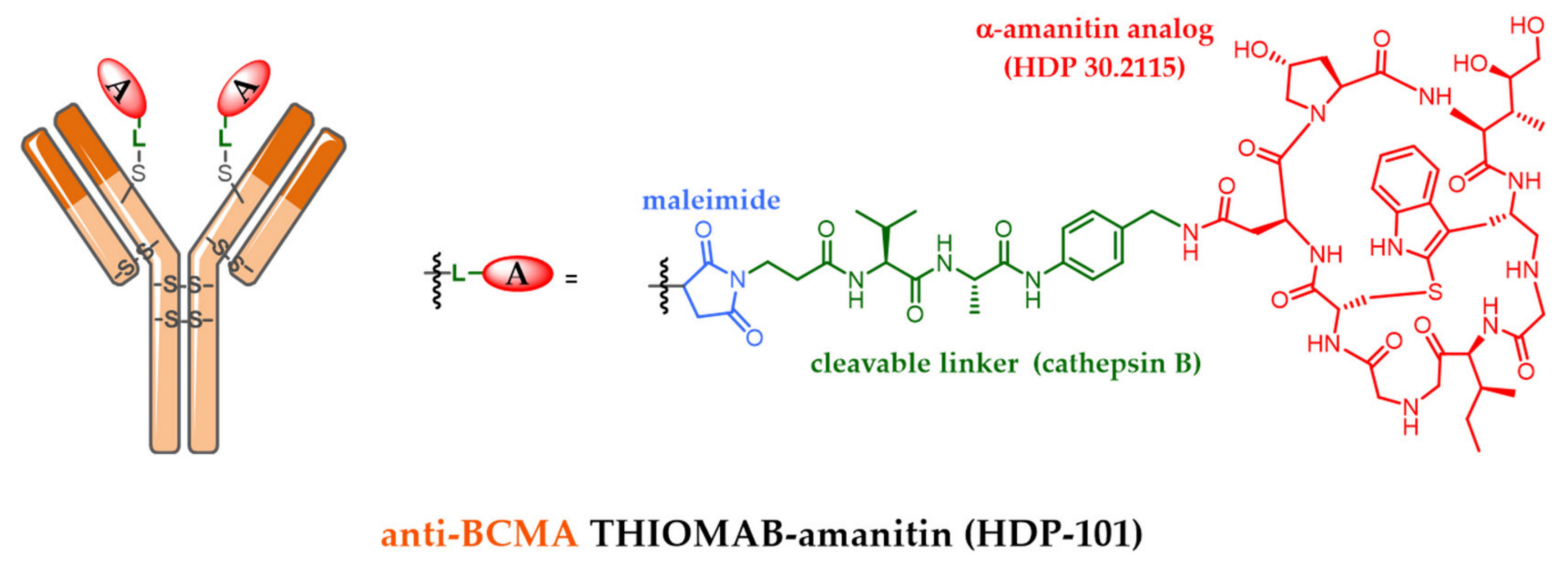
HDP-101 formula, ADC with a DAR 2, resulting from the site-specific conjugation of an α-amanitin analog (HDP 30.2115) onto an anti-BCMA Thiomab via a cathepsine B-sensitive linker.

As an alternative to the previously described ADCs, Heidelberg Pharma developed a proprietary technology called ATAC (for antibody targeted amanitin conjugate) [93,94] based on the conjugation of amatoxins, which are strong RNA polymerase II inhibitors (new mechanism of action for an ADC payload). Alpha- and beta-amanitin are two well-known amatoxins, identified in the mushroom *Amanita phalloides* more than forty years ago. HDP-101 (Figure 11) is the most advanced ATAC. HDP-101 results from the site-specific conjugation of HDP 30.2115, a stabilized analog of α-amanitin, onto a Thiomab (designed by Genentech) targeting the B-cell maturation antigen (BCMA, CD269) through a cathepsin B-sensitive linker, to produce an ATAC with a DAR of 2. BCMA has emerged as a very selective target of choice for the treatment of multiple myeloma [95]. In vitro, HDP-101 was tested on various multiple myeloma cells taken from patients, including non-dividing cells or cells with a low concentration of BCMA antigens (down to 270 copies per cell) [96]. In all cases, the observed IC_50_ of the ATAC HDP-101 was in the picomolar range. Due to the hydrophilicity of the amatoxins, the observed IC_50_ of the ATAC HDP-101 is 20,000-fold higher (around 100 pM) than the IC_50_ of its free amatoxin HDP 30.2115. No toxicity was observed on non-BCMA expressing cells. Therefore, the ATAC mechanism is only based on the internalization of the ATAC-Ag complex followed by its lysosomal degradation in BCMA-positive tumor cells. In vivo, in various xenograft models of multiple myeloma, HDP-101 led to complete tumor remission in mice, and safety studies identified a very favorable therapeutic window in mice and monkeys. ATACs are a very promising class of compounds, still in a preclinical phase, although their chemistry manufacturing control (CMC) process has been successfully carried out.

### 5.6. Combined Strategies beyond Dogmas: Pivotal Phase II or Phase III ADC

The development of first and especially second-generation ADCs has been associated with many dogmas, which many studies have considered as rules to follow in order to develop ADCs with better chances of success. Among these dogmas, we can cite targeting of an Ag not expressed in a ubiquitous manner, with a high overexpression level and internalizing, in particular to allow the intracellular release of the cytotoxic via a well-designed linker. The cleavable linker should be more stable than those sensitive to an acidic or reducing medium. The cytotoxic must have a potency at least like auristatins and maytansines to be used in ADC. Finally, the only format of interest for the antibody was IgG to guarantee a long half-time life and possibly the conservation of the effector activity from the parent mAb to the ADC. We will see that recent ADCs combine several innovations (among the target, format, release system and cytotoxic action mechanism), but they also shake up one or more dogmas with success.

**Figure 12:**
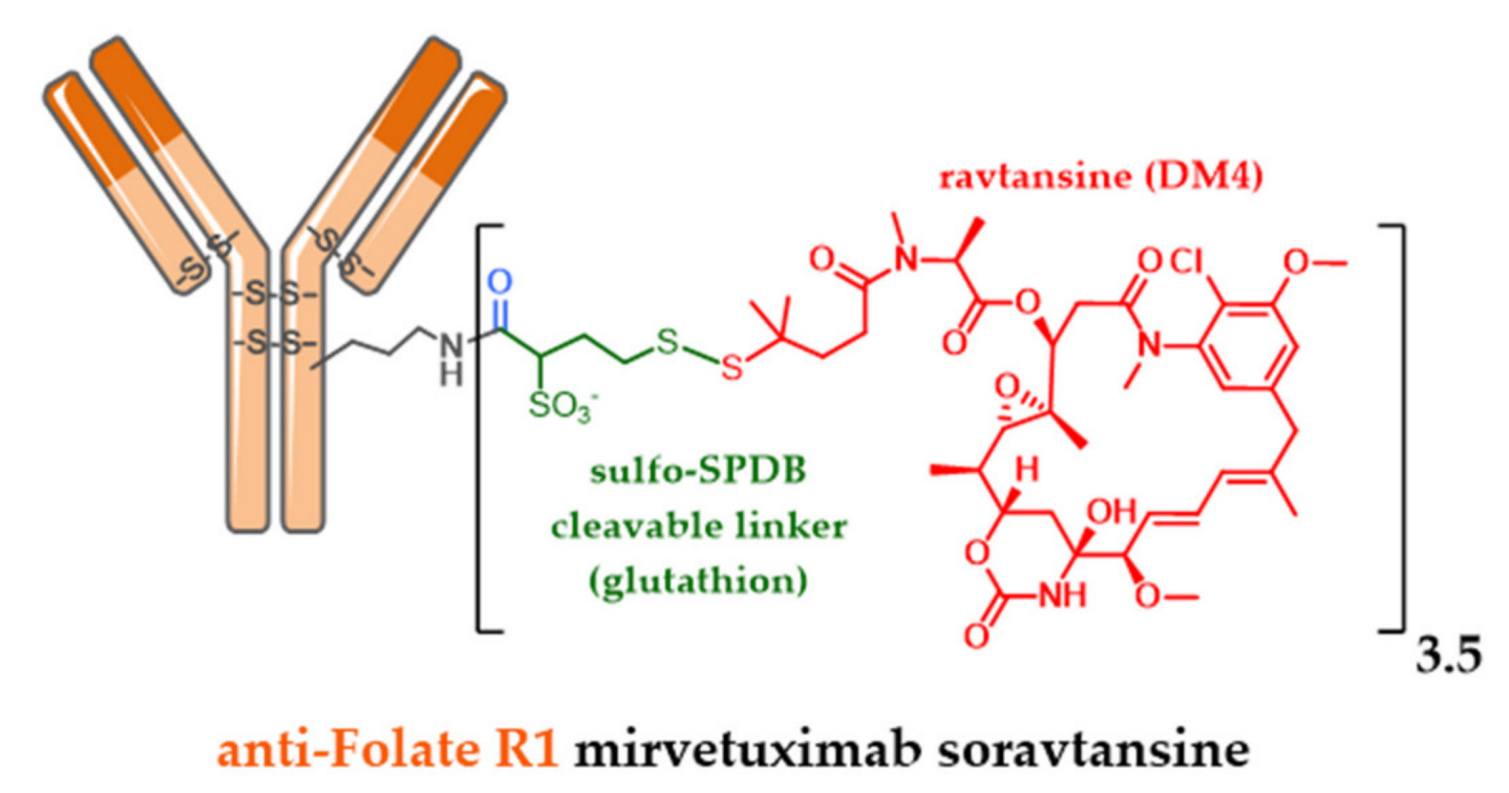
Formula of mirvetuximab soravtansine, antifolate R1 antibody conjugated to DM4 via a non-cleavable linker.

Among them, Immunogen succeeded in developing mirvetuximab soravtansine (Figure 12) [97,98,99]. This ADC is rather surprising because the release system in reducing conditions (sensitive to glutathione) was too sensitive (because of being unstable in plasma in the early development stage) and the corresponding linker was associated with a lack of success during the development of the ado-trastuzumab emtansine. Mirvetuximab soravtansine therefore results from the bioconjugation of DM4 to an antifolate antibody R1, via a cleavable linker in a reducing medium (rich in glutathione), optimized by the presence of two methyl groups in the alpha of the disulfide bond and by the presence of a sulfonyl group improving the hydrophilicity of the linker and therefore the bioconjugation of DM4. This ADC is currently being tested against epithelial ovarian cancer in a phase III clinical study.

In a disconcerting manner, despite the development of many technologies allowing a site-specific bioconjugation of a mAb to result in a more homogeneous ADC with an improved therapeutic index (10 in clinical study and more than 40 in preclinical), none of them has yet been validated by the approval of a homogeneous ADC.

**Figure 13:**
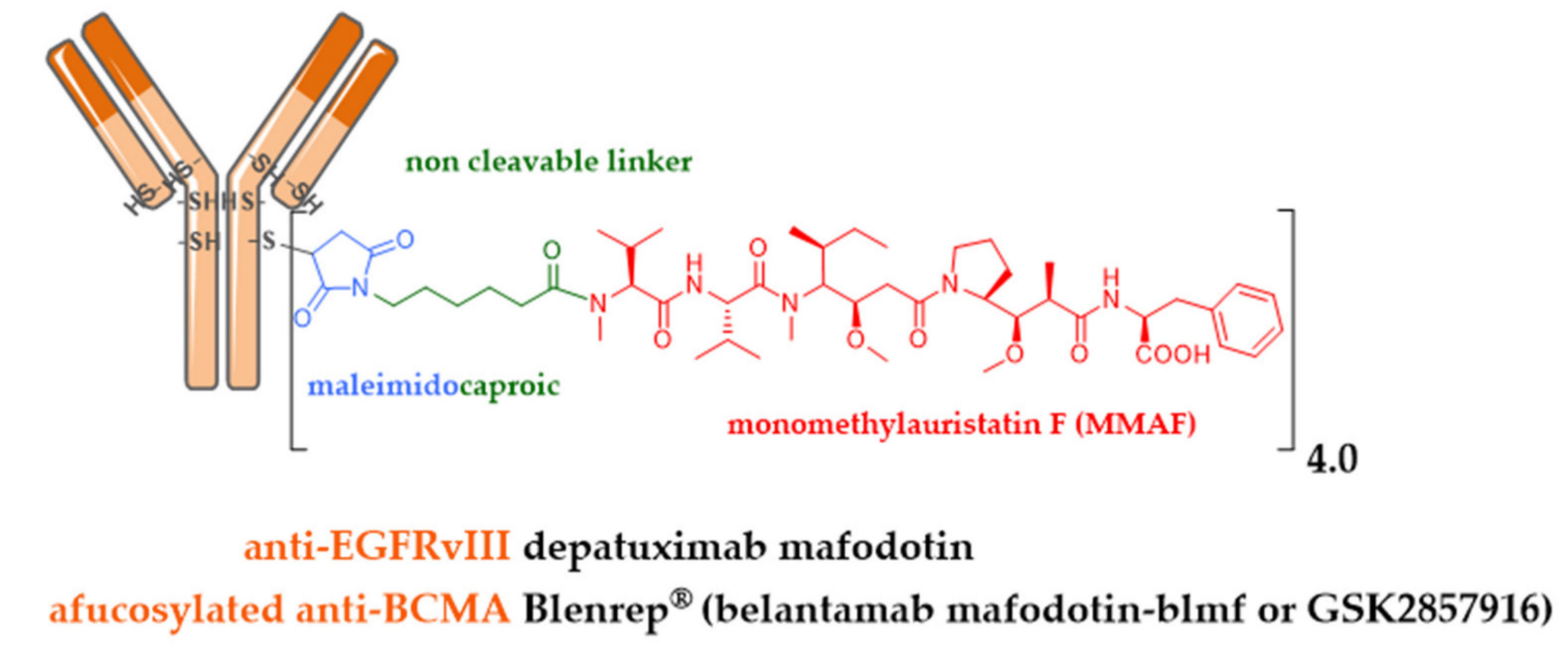
Formula of depatuximab mafodotin and Blenrep^®^ (belantamab mafodotin-blmf), antibodies conjugated to MMAF via a non-cleavable linker.

EGFR (epithelial growth factor receptor) is ubiquitously expressed in epithelial cells in general and in the skin. Therefore, designing an ADC targeting EGFR did not seem obvious. However, Abbvie designed ADC ABT-414 by stochastically bioconjugating MMAF via a non-cleavable maleimide linker on a mAb targeting a unique epitope of EGFR overexpressed in tumors, EGVRvIII (Figure 13). The cytotoxic payload is MMAF, an analogue of MMAE used in Adcetris^®^ and Polivy^®^, but which has been optimized by Seattle Genetics to be used with a non-cleavable caproic linker (which is not possible with MMAE). Depatuximab mafodotin was still recently tested against glioblastoma in a phase III clinical study [100,101,102] but unfortunately these clinical trials were also stopped in 2019.

On the other hand, belantamab mafodotin (GSK2857916), developed by GSK, using the same conjugation technology (maleimide + MMAF) on an afucosylated anti-BCMA IgG1 antibody (Figure 13), has successfully finished a pivotal phase II clinical study against multiple myeloma [103], for patients whose disease has progressed despite prior treatment with an immunomodulatory agent, proteasome inhibitor and anti-CD38 antibody. Following a biologics license application (BLA) filled early in 2020, Blenrep^®^ has just been approved by the FDA as well as by the EMA, as a first-in-class anti-BCMA therapy against multiple myeloma.

Despite Ag specific targeting, ADCs are sometimes associated with high toxicity, specific or not to their target. This toxicity is linked to several mechanisms leading to the uncontrolled early release of the potent payload carried by the ADC outside the tumor. Unfortunately, in 2019, the ADCs area of research has not yet found the magic bullet that Paul Ehrlich dreamed of at the start of the 20th century. Given this observation, some companies have successfully turned to the development of original ADCs using less potent cytotoxic agents than MMAE or DM1, with new mechanisms of action to fight resistance to tubulin polymerization inhibitors. These ADCs also have release systems that are not necessarily specific for intracellular conditions and target original or unconventional targets.

**Figure 14:**
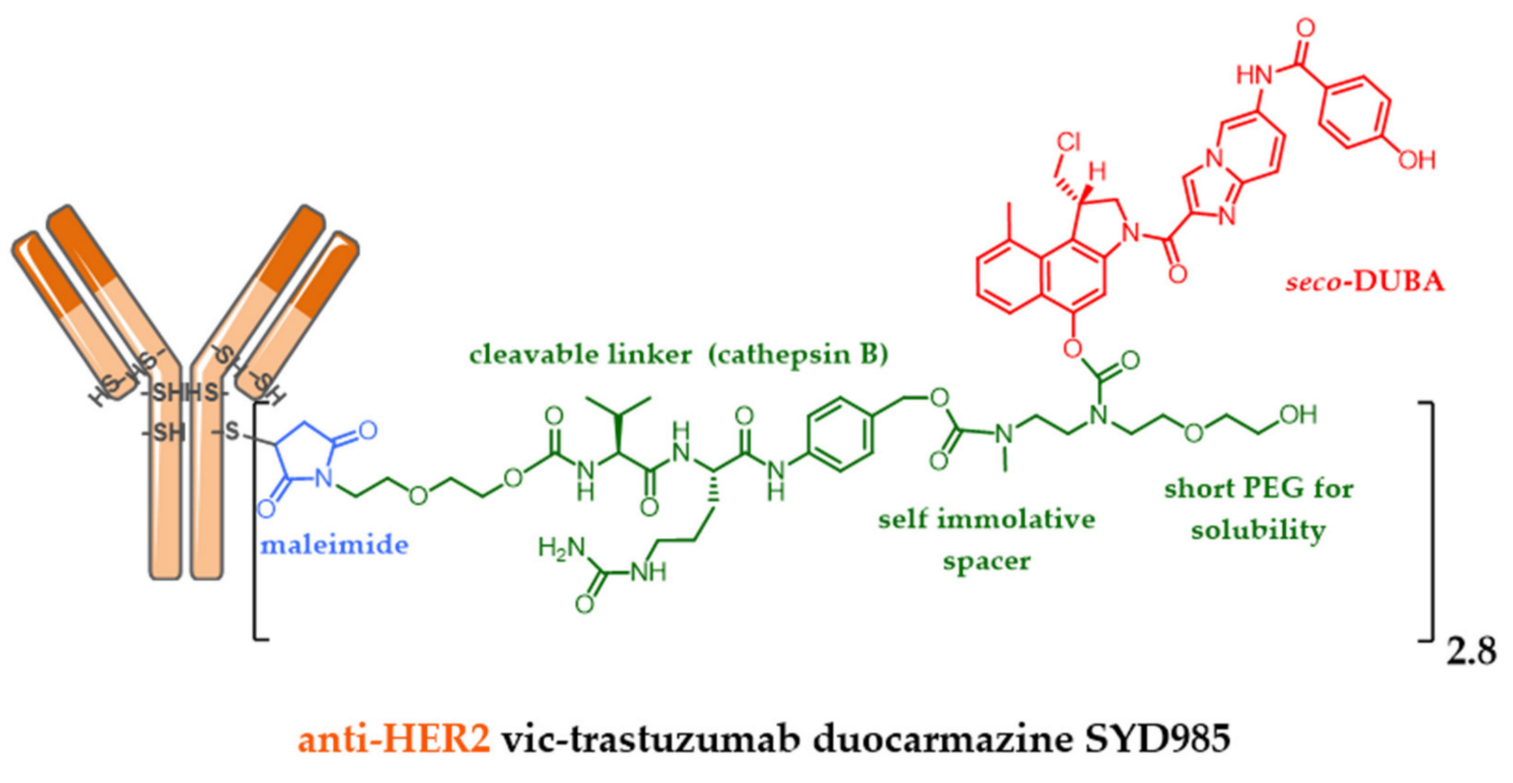
SYD985 formula, anti-HER2 trastuzumab conjugated to *seco*-DUBA via a cleavable linker sensitive to cathepsin B.

For example, Byondis B.V. (formerly Synthon Biopharmaceuticals) developed trastuzumab duocarmazine (SYD985) [104], an ADC with an average DAR of 2.8, resulting from the combination of an anti-HER2 mAb and a duocarmycin precursor (seco-DUBA), linked by a cleavable linker sensitive to cathepsin B, optimized by the presence of two small pegylated units to ensure better solubility (and therefore better conjugation) of the linker-payload moieties (Figure 14). SYD985 is currently being tested against T-DM1 in a phase III clinical study in HER2-positive metastatic breast cancer.

### 5.7. Successful Iteration beyond Dogmas: Recent Approval of Third Generation Enhertu^®^ and Trodelvy^®^

**Figure 15:**
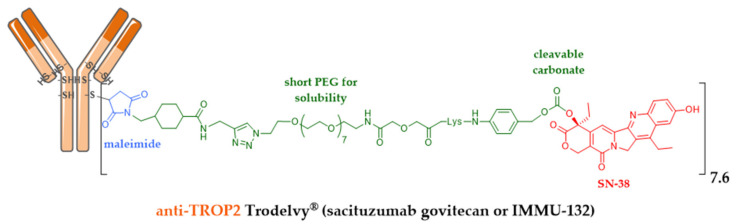
Trodelvy^®^ (sacituzumab govitecan or IMMU-132) formula, ADC with a DAR 7.6, resulting from anti-TROP-2 antibody conjugation to SN-38 via an acid-sensitive cleavable linker.

Among the many companies developing ADCs, Immunomedics designed surprising ADCs by making a triple bet: to build an ADC targeting a slightly overexpressed target, using a system mixing intra- and extracellular release and a less potent payload than those conventionally used in ADC (MMAE and DM1). Sacituzumab govitecan (IMMU-132, Figure 15) is an anti-TROP-2 [105] mAb conjugated to SN-38 (the active metabolite of irinotecan) via a cleavable maleimide linker (acidity) with a short pegylated unit [106]. The FDA-approval of this ADC was delayed and reached in April 2020, after a second BLA (biologics license applications) process was necessary to solve certain CMC issues following a successfully carried out phase III study. Bet successful for Immunomedics: the accomplishment is all the more impressive since this ADC is indicated in refractory or resistant triple negative breast cancer (TNBC) against which there was no treatment until the FDA-approval of Trodelvy^®^ in April 2020 [107]. Another interesting feature of this ADC: the optimization of the linker structure including a pegylated unit led to this ADC with a high DAR of 7.6 [105], without compromising its tolerance or efficiency. DAR 4 has long been considered as optimal, but this statement is now only true for the known approved ADCs with a second generation linker carrying DM1 or MMAE as the payload. IMMU-132 is a very interesting case study demonstrating that the optimal DAR of an ADC will depend on many parameters, mainly the hydrophilic nature of the linker and the grafted payload.

**Figure 16:**
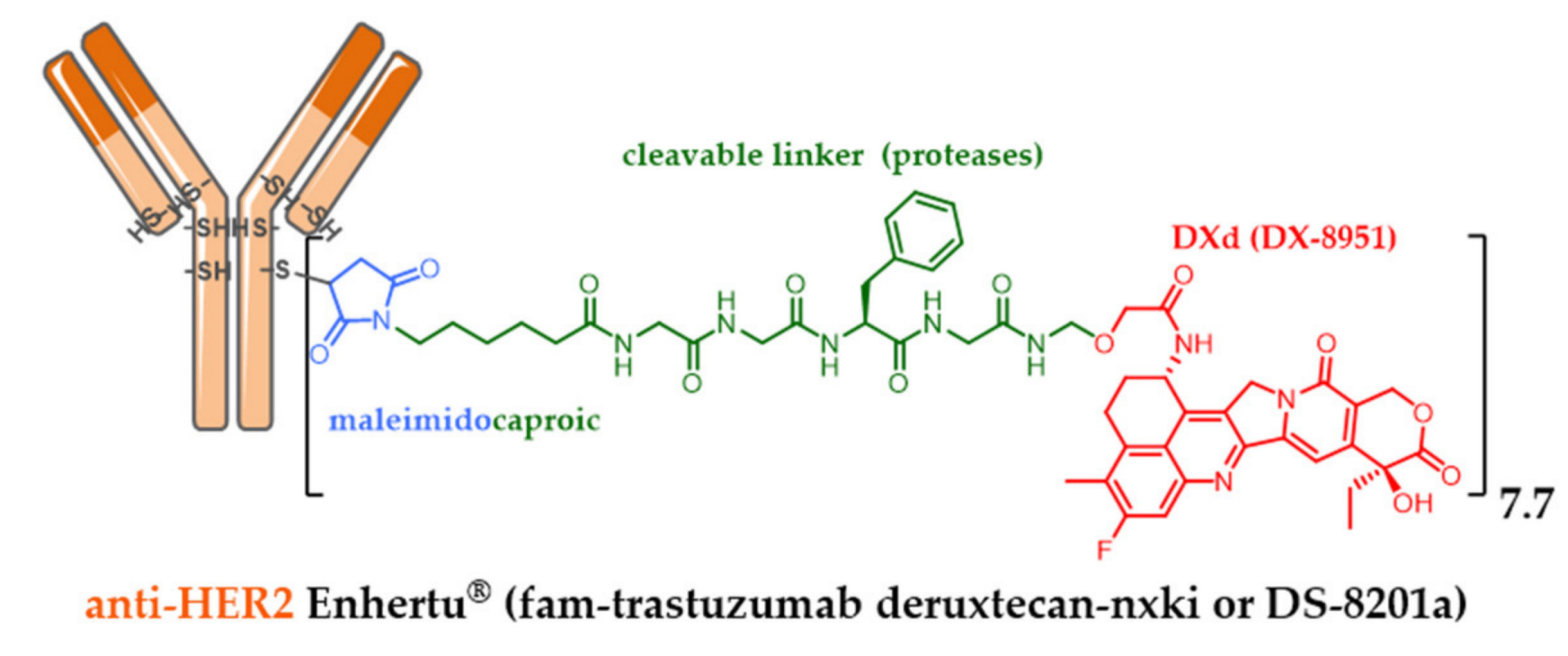
Enhertu^®^ (fam-trastuzumab deruxtecan-nxki or DS-8201a) formula, ADC with a DAR 7.7, resulting from anti-HER2 trastuzumab conjugation to exatecan DX-8951 via a linker sensitive to proteolysis.

Similarly, in order to conjugate an irinotecan derivative with a meticulously designed linker, the Japanese company Daiichi Sankyo developed DXd (exatecan or DX-8951). DXD is a cytotoxic agent 10-fold more active than SN-38 in vitro on cancer cells. DXD has a better safety profile with an optimized solubility, able to elicit a bystander killing effect [25] to kill neighboring cancer cells, which is an advantage in the heterogeneous tumor, but with a short half-life to avoid off-target toxicity. Bioconjugation of DXd onto the anti-HER2 trastuzumab cysteine residues via a maleimide linker sensitive to proteolysis made it possible to obtain the conjugate fam-trastuzumab deruxtecan-nxki (DS-8201a) with a homogeneous DAR of 7.7 (Figure 16) [108,109]. Despite its high DAR, Daiichi Sankyo’s DS-8201a was very well tolerated in rats and monkeys, and very stable in plasma (2.1% of DXD release after 21 days of incubation, in comparison to the release rate of T-DM1, which was 18.4% after only 4 days, despite the use of a non-cleavable linker). The use of a high DAR compound is remarkable as it contradicts the widely established principle that high DAR conjugates are unlikely to be good candidates due to poor pharmacokinetic profiles. These achievements were possible after testing many linkers. The chosen enzyme-sensitive linker encompasses a tumor-selective GGFC cleavable linker and an amino methylene SIS with a reduced hydrophobicity and a better plasma stability in comparison to the classical PAB SIS. Once DS-8201a is internalized, its linker is selectively cleaved by lysosomal proteases after the GGFG sequence to release a temporary DXD hydrolysate, which SIS amino-methylene is subsequently hydrolyzed to ammonia and formaldehyde to free DXD, triggering cell death.

While Kadcyla^®^ (T-DM1) is known to have in vitro efficacy only against HER2-positive cells with a high HER2 expression level, DS-8201a was effective in the pancreatic Capan-1 cell line with low HER2 expression and in the T-DM1 refractory JIMT-1 HER2-positive breast cancer cell line. With a DAR of 8 and a payload able to elicit the bystander killing effect, DS-8201a can effectively deliver DXD in a heterogeneous tumor in vivo and exhibit a high therapeutic effect [110]. DS-8201a was successfully tested against T-DM1 in a phase III clinical study in metastatic HER2-positive breast cancer last year. Enhertu^®^ was finally approved by the FDA in late December 2019.

## 6. Indications of ADCs

### 6.1. Combinations with Conventional Chemotherapy

Many studies are currently exploring combinations of Adcetris^®^ or Kadcyla^®^ with conventional chemotherapy. The objective may be here to replace an antitubulin agent with an ADC coupled with an antitubulin agent and likely to cause less toxicity, for example by replacing vincristine by Adcetris^®^ in the treatment of certain lymphomas (NCT01777152). This could be particularly useful in fragile patients or those whose comorbidities preclude the use of conventional agents. Another objective may be to strengthen the activity of an established combination whose mechanism of action is different. As an example, Adcetris^®^ is used with a combination of cisplatin, dexamethasone and cytarabine for the treatment of Hodgkin’s disease [111].

### 6.2. Adjuvant, Maintenance or Consolidation Treatments

A growing number of patients are in remission from their cancers by a first line treatment, nevertheless without being in complete remission or cured. Several situations currently require adjuvant treatment (when the disease is not detectable) or maintenance (when the patients are in partial response). Kadcyla^®^ has thus shown its superiority over trastuzumab, its unconjugated equivalent, in the randomized study KATHERINE (NCT01772472) as an adjuvant treatment in patients retaining residual breast or lymph node disease after neoadjuvant treatment with a decrease in 50% of the risk of local recurrence or death [112]. It is likely that other pathologies, in which maintenance treatment with naked mAbs have already proven their effectiveness, such as certain types of malignant lymphomas for example, can also benefit from the administration of ADC following a first line treatment.

### 6.3. Combinations of ADC and Immune Checkpoint Inhibitors

The value of combining cytotoxic chemotherapy with immune checkpoint inhibitors (ICPI) such as anti-PD1 and anti-PDL1 is currently the subject of numerous clinical studies [113]. In addition to the complementary mechanisms of action, the possibility of increasing the immunogenicity of tumors through immunogenic death induced by chemotherapy constitutes a strong argument for the association of certain cytotoxic agents with immunotherapies. The combination of ADC with ICPIs therefore appears to be a logical step, especially in patients who are already heavily pretreated and who want to avoid the systemic toxicity of chemotherapy. A phase 1/2 study of the combination of Adcetris^®^ and nivolumab, an antibody directed against PD1, in patients with relapsed or refractory Hodgkin’s disease showed a response rate of 82%, including 61% complete responses [114]. These results were then confirmed in the Checkmate 205 study with responses in more than two thirds of the patients [115].

In general, ADCs have been shown to be effective in relapses and more recently in adjuvant situations in HER2-positive breast cancer. The positioning of these agents in the future will depend on several factors including the importance of the advantages brought compared to conventional chemotherapy (either in terms of toxicity or antitumor activity), the available therapeutic alternatives and the cost of patient care. ADCs are still a recent family of compounds. The approved agents are based on highly toxic payloads with mechanisms of action similar to those of conventional cytotoxic chemotherapy. The development of new ADCs based either on conventional agents such as sacituzumab govitecan, an antibody whose conjugate is the active metabolite of irinotecan [116] or on payloads with the original mechanisms of action, could also have a significant impact on the clinical use of ADC.

## 7. Quick Overview of ADC beyond Oncology

The success of ADCs as anticancer agents encouraged many researchers and pharmaceutical companies to open the frontier of their application domains beyond oncology. Indeed, recently several ADCs have been developed as immunomodulatory agents or against infectious diseases [117].

### 7.1. ADC as an Immunomodulatory Agent

**Figure 17:**
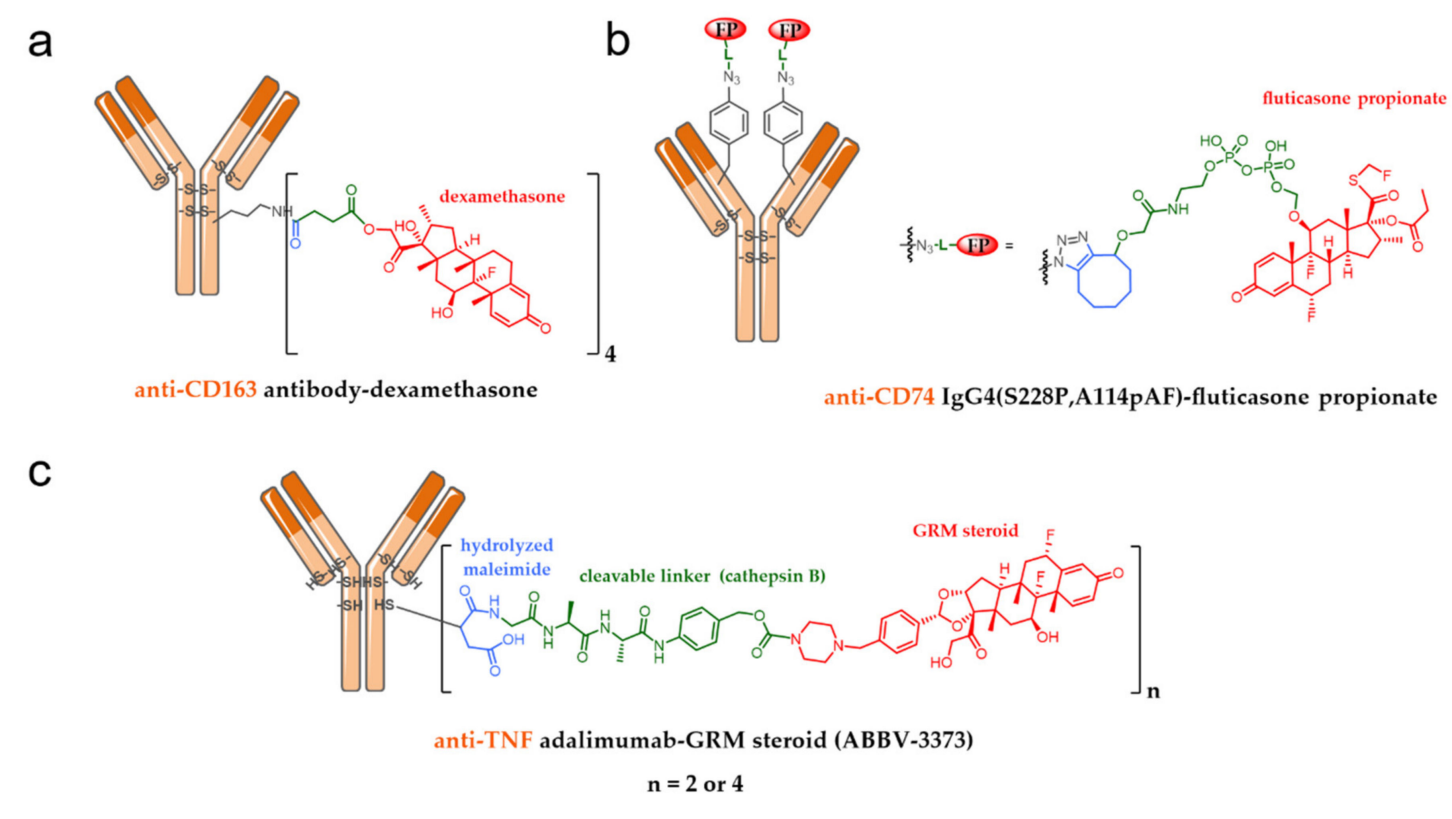
(**a**) Formula of an ADC resulting from the conjugation of dexamethasone onto an anti-CD163 mAb, with a DAR of 4. (**b**) Formula of an ADC resulting from the site-specific conjugation of fluticasone propionate onto an anti-CD74 IgG4 mutated mAb, with a homogeneous DAR of 2. (**c**) Formula of an ADC resulting from the conjugation of a dexamethasone analog onto anti-TNF-α adalimumab, with a DAR of 2 or 4.

Glucocorticoids (GC) are potent anti-inflammatory drugs but are associated with systemic toxicity leading to serious side effects including but not limited to immunosuppression and metabolic disorders. GC anti-inflammatory effects are very complex but mediated the suppression of tumor-necrosis factor-α (TNF-α) and other cytokines release by macrophages. To increase dexamethasone activity and limit its toxicity, an anti-CD163 dexamethasone-conjugate with a DAR of 4 was designed (Figure 17a) and biologically tested. This anti-CD163 ADC, targeting macrophages, elicited reduced lipopolysaccharide-induced secretion of TNF-α in vitro. Moreover this ADC was about 50-fold more active in vivo than the nonconjugated dexamethasone in a Lewis rat model, without exhibiting any sign of a systemic effect as opposed to the unconjugated drug [118,119].

To reduce dose-limitation due to on-target off-site toxicity, fluticasone propionate (another GC) and one of its analogs were respectively site-specifically conjugated onto an anti-CD74 mAb targeting B-cells. The IgG4 antibody was stabilized with the S228P mutation, and two *para*-azido phenylalanine (pAF) residues were incorporated at position 114, to design ADCs with a DAR of 2 (Figure 17b). While the fluticasone propionate-conjugate was associated to a lack of activity due to unwanted payload release, the ADC resulting from the conjugation of a GC receptor agonist analog of fluticasone propionate to the anti-CD74 IgG4 exhibited cell-intrinsic activity in human B cells [120].

The most advanced ADC including a GC in clinical trials is ABBV-3373 (Figure 17c), in a phase 2a study, developed by Abbvie against rheumatoid arthritis. ABBV-3373 is an ADC resulting from the conjugation of a proprietary glucocorticoid receptor modulator (GRM), derived from dexamethasone, on the anti-TNF-α adalimumab, with a DAR of 2 or 4, according to the bioconjugation process. This latter was optimized using a hydrolyzed maleimide as the bioconjugation moiety, to induce better stability in plasma circulation, by avoiding deconjugation via a retro-Michael reaction. The mechanism of action of ABBV-3373 is a targeted uptake at the surface of immune cells expressing transmembrane TNF, followed by internalization and lysosomal GC release. The released GC then activates the GR pathway and provokes an anti-inflammatory cascade in the nucleus. This targeted mechanism dampens the systemic side effects observed with GC steroids [121].

**Figure 18:**
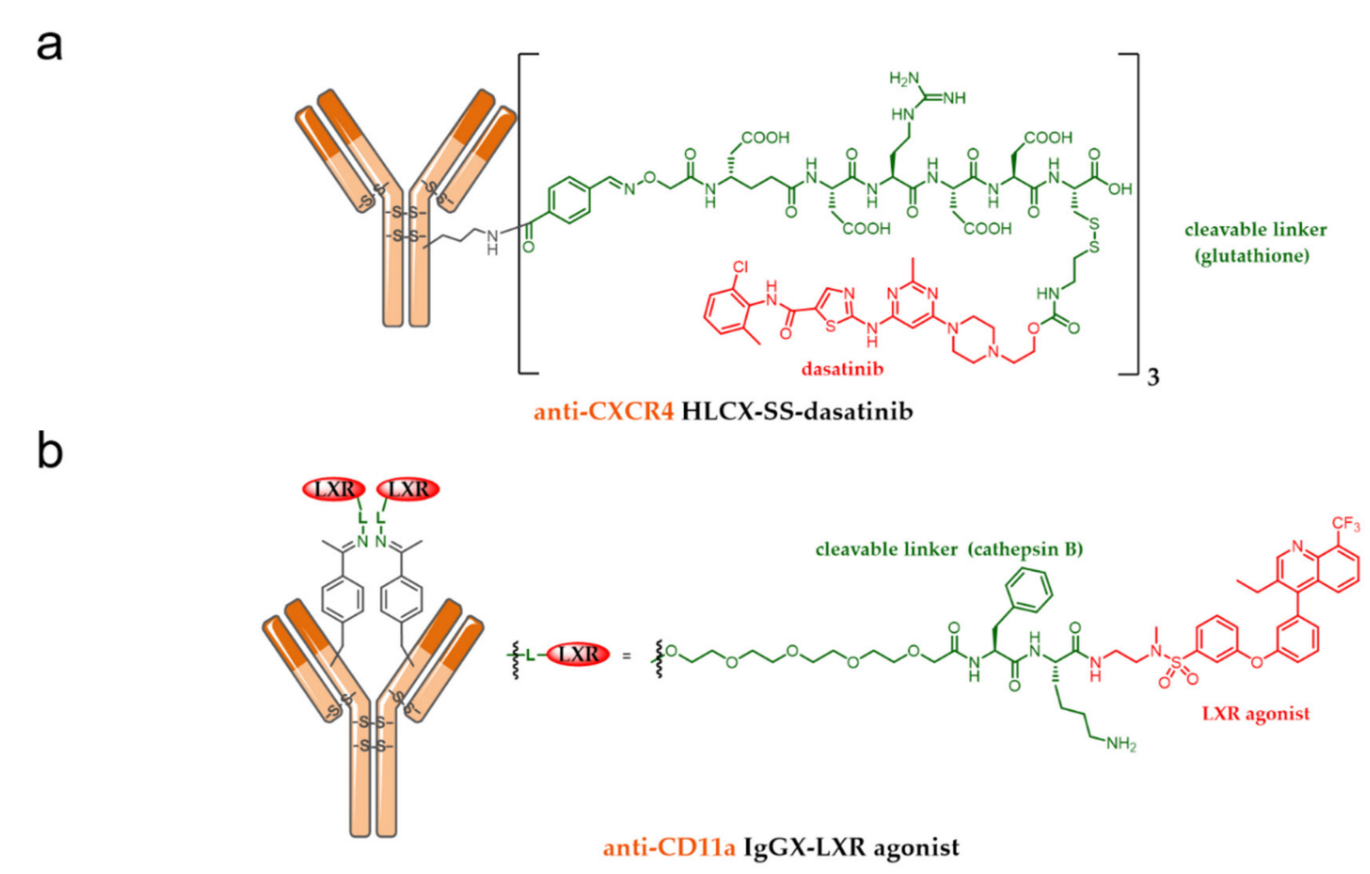
(**a**) Formula of an ADC resulting from the stochastic conjugation of dasatinib onto an optimized anti-CXCR4 mAb (HLCX) through a gluthathione sensitive linker, with an average DAR of 3. (**b**) Formula of an ADC resulting from the site-specific conjugation of a LXR agonist onto an anti-CD11a mutated mAb, with a homogeneous DAR of 2.

Kazane et al. from the California Institute for Biomedical Research developed two immunosuppressive ADCs. The proof of concept of this approach demonstrated that the conjugation of dasatinib onto an anti-CXCR4 mAb was able to generate an immunosuppressive ADC carrying a payload that is not a GC [122]. Dasatinib is an orally bioavailable potent kinase inhibitor (IC_50_ < 1 nM) targeting Bcr-Abl tyrosine kinase and Src family kinases including Lck and Fyn, playing a key role in T-cell receptor activation. While dasatinib is clinically used to treat Bcr-Abl-dependent chronic myelogenous leukemia, its lack of selectivity is associated with serious side effects undermining its development as an immunosuppressant drug. To reach that goal, dasatinib was stochastically conjugated onto a modified anti-CXCR4 mAb (HLCX). HLCX was optimized to bind to the loop outside the ligand-binding pocket of CXCR4 at the surface of human T lymphocytes, to favor an efficient internalization. Two linkers were designed for the ADC conception: the first one is the glutathione-sensitive linker (for ADC HLCX-SS-dasatinib, Figure 18a) while the second one is uncleavable (for ADC HLCX-dasatinib). Each ADC had an average DAR of 3. HLCX-SS-dasatinib was shown to be two-fold more potent than HLCX-dasatinib in T-cell activation assays, by suppressing IL-2, TNF-α and IFN-γ secretion.

In another approach, a liver X receptor (LXR) agonist, able to suppress inflammation, was site-specifically conjugated onto an anti-CD11a mAb (IgGX) targeting monocytes/macrophages for the treatment of atherosclerosis. Two *para*-acetylphenylalanine (pAcPhe) residues were site-specifically incorporated into alanine 121 of IgGX, to yield the conjugate IgGX-CatB-LXR with a DAR of 2 (Figure 18b) after reaction with the terminal aminooxy group of the cathepsin B-sensitive heterobifunctional linker carrying the LXR agonist. While LXRs are found in monocytes/macrophages and in kidney, liver and intestine, the conjugate IgGX-CatB-LXR selectively activated LXR in THP-1 monocytes/macrophages but not in HepG2 hepatocytes in vitro, with 3-fold greater potency than a known LXR agonist [123]. This approach was designed to avoid excessive lipogenesis due to on-target adverse effects in the liver, an in vivo study is currently ongoing to prove this statement. These strategies show that antibody-targeting offers significant potential for rescuing existing and new dose-limited drugs outside the field of oncology and may lead to a new class of selective immunosuppressive drugs with improved safety profiles.

### 7.2. Antibody-Antibiotic Conjugate

*Staphylococcus aureus* is the leading cause of bacterial infections while antibiotic resistance is spreading worldwide. Some *S. aureus* strains are described to survive standard antibiotic treatment by ‘hiding’ in host cells like phagocytes, with reduced susceptibility to vancomycin and to resistance to linelozid, daptomycin and all β-lactam antibiotics, with the emergence of methicillin-resistant *S. aureus* (MRSA).

**Figure 19:**
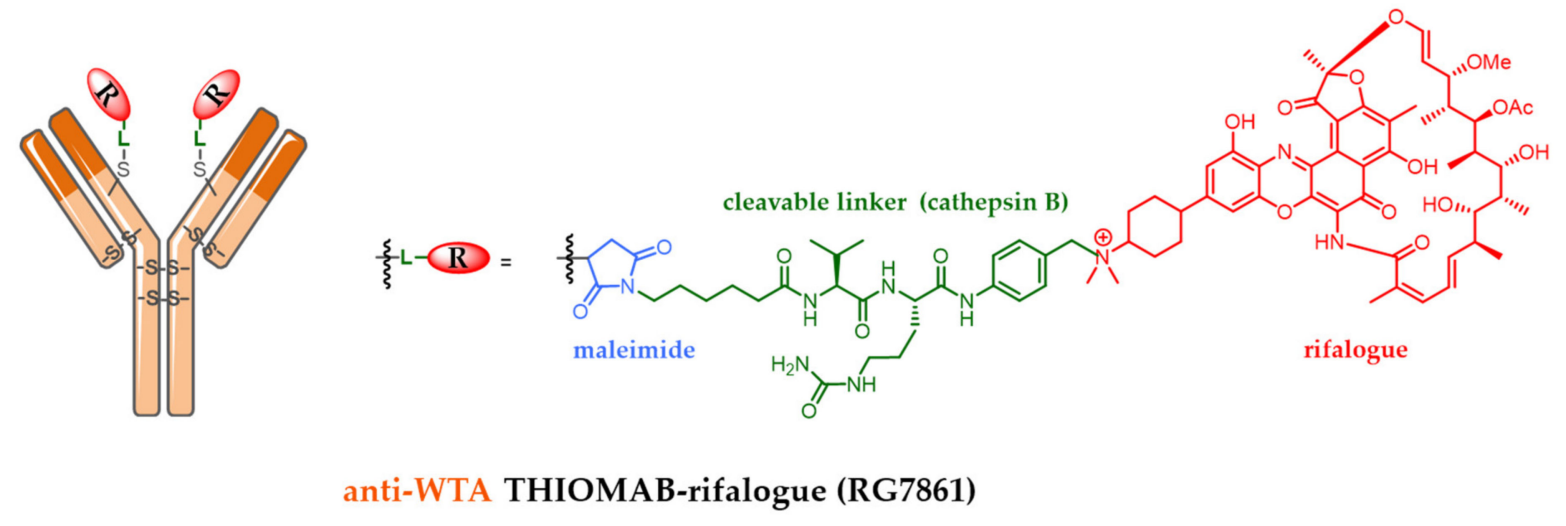
RG7861 formula, ADC with a DAR 2, resulting from the site-specific conjugation of rifalogue onto an anti-wall-teichoic acid (WTA) Thiomab via a cathepsine B-sensitive linker, targeting the surface of *Staphylococcus aureus*.

To circumvent the lack of intracellular bacteria targeting associated with actual treatments, Genentech decided to develop an antibody–antibiotic conjugate (AAC), designed with a carefully selected antibody, linker and antibiotic, to achieve better activity than vancomycin for the treatment of bacteremia in vivo [124,125]. To achieve this goal, Genentech developed a thiomab directed against pathogen-specific wall-teichoic acids (WTA) present at the bacteria surface. While several antibiotics were tested, only rifalogue, a rifamycin derivative, was selected for its ability to accumulate intracellularly and for its unaltered highly potent bactericidal activity at low pH. Rifalogue was site-specifically conjugated onto the anti-WTA Thiomab through a cathepsin B-sensitive linker to produce an AAC with a DAR of 2 (Figure 19).

The mechanism of this AAC was its ability to target and fix extracellular *S. aureus. S. aureus* opsonization leads to its Fc-mediated internalization inside a host phagocyte. This is followed by selective rifalogue release by degradation of the AAC linker in the proteolytic environment of phagolysosome, leading to intracellular bacteria killing.

Interestingly, the same strategy was applied to rifampicin, but the resulting AAC was not active in vivo, highlighting and confirming the fact that, like for ADC in oncology, each AAC component should be carefully selected to achieve the desired therapeutic effect.

This impressive work could allow some potent antibiotic drugs, forsaken in clinical trials due to host toxicities and unfavorable pharmacokinetic profiles, to be evaluated again as judiciously designed immunoconjugates.

## 8. Conclusions and Perspectives

ADCs represent today a recent success of an old approach in chemotherapy targeting cancer. The classic internalizing ADCs currently used in clinics are designed to specifically deliver powerful cytotoxic agents to the targeted Ag (+) cancer cells, to eliminate only Ag (+) cancer cells (non-cleavable linker) or all tumor cells including both Ag (+) and Ag (−) cancer cells (cleavable and cytotoxic linker, rather hydrophobic). Over the past decade, ADCs have been improved by the choice of better cytotoxic agents, bioconjugation methodologies, better chosen targeted antigens and optimized antibody engineering.

However, despite their sophisticated design, ADCs are still associated with several limitations (for example, limited solid tumor penetration and toxicity) and the emergence of resistance mechanisms. To overcome these limitations, new antibody formats, new delivery systems, non-internalizing antigenic targets, new cytotoxic agents and site-specific bioconjugation methods have been studied to advance the development of ADCs. Unfortunately, many innovations have not yet been validated for use in clinical protocols, since the resulting ADCs are still in a preclinical or clinical study, and a small number have reached pivotal clinical phase II or phase III.

In conclusion, this field of research offers many encouraging prospects, especially when ADCs are combined with conventional chemotherapy or checkpoint inhibitors, in order to better potentiate their effects. In the last decade, the search for novel targets and the use of judiciously chosen payloads, with a well-designed release mechanism, have successfully opened the ADC field of applications beyond oncology, with ADC reaching up to phase 2a clinical trials. Some of these ADCs can fight resistant bacteria. Hopefully, this research may allow forsaken drugs from the past to shine again as new conjugated drugs with better pharmacological profiles and efficacy, and allow ADCs to get a little closer to the magic bullet imagined by Paul Ehrlich at the beginning of the 20th century.

## Figures and Tables

**Table 1:** Antibody–drug conjugates (ADCs) approved by the Food and Drug Administration (FDA), in advanced clinical trials (Phase III or pivotal phase II) or recently stopped.

Company	ADC (Cytotoxic)	Isotype and Target	Indication/Approval Date (Trade Name)/Clinical Status
Pfizer	gemtuzumab ozogamicin (CAL)	IgG4 CD33	2000–2010/2017 AML (Mylotarg^®^)
Seattle Genetics	brentuximab vedotin (AUR)	IgG1 CD30	2011 ALCL and Hodgkin lymphoma (Adcetris^®^)
Roche	trastuzumab emtansine (MAY)	IgG1 HER2+	2013 metastatic HER2+++ breast cancer (Kadcyla^®^) **
Pfizer	inotuzumab ozogamicin (CAL)	IgG4 CD22	2017 ALL and CLL (Besponsa^®^)
Roche	polatuzumab vedotin (AUR)	IgG1 CD79b	2019 DLBCL (Polivy^®^)
Seattle Genetics	enfortumab vedotin (AUR)	IgG1 Nectin 4	2019 urothelial cancer (Padcev^®^) **
Daiichi Sankyo	trastuzumab deruxtecan (EXA)	IgG1 HER2+	2019 metastatic HER2+++ breast cancer (Enhertu^®^) **
Immunomedics	sacituzumab govitecan (IRI)	IgG1 TROP-2	2020, metastatic TNBC (Trodelvy^®^) **
GSK	belantamab mafodotin (AUR, MMAF)	IgG1afuc BCMA	2020, multiple myeloma (Blenrep^®^)
Byondis *	trastuzumab duocarmazine (DUO)	IgG1 HER2+	Ph 3 metastatic HER2+++ breast cancer (vs T-DM1) **
Bio-Thera	BAT8001 (MAY)	IgG1 HER2+	Ph 3 breast cancer HER2+++ (vs T-DM1) **
ImmunoGen	mirvetuximab soravtansine (MAY)	IgG1 Folate R1	Ph 3 epithelial ovarian cancer **
Sanofi (IMG)	SAR408701 (MAY, DM4)	IgG1 CEACAM5	Ph 3 metastatic small cell lung cancer **
ADC-Therapeutics	loncastuximab tesirine (PBD)	IgG1 CD19	Piv. Ph2 diffuse large B-Cell lymphoma (BLA 2H2020)
ADC-Therapeutics	camidanlumab tesirine (PBD)	IgG1 CD25	Pivotal Ph2 Hodgkin lymphoma
Seattle Genetics	vadastuximab talirine (PBD)	IgG1 CD33	Ph 3 AML (stopped, 2017)
AbbVie	rovalpituzumab tesirine (PBD)	IgG1 DLL3	Ph 3 small cell lung cancer (stopped, 2019) **
AbbVie	depatuxizumab mafodotin (AUR)	IgG1 EGFRvIII	Ph 3 glioblastome (stopped, 2019) **

* formerly Synthon Biopharmaceuticals, ** ADC targeting solid tumor.
